# Proximal telomeric decompaction due to telomere shortening drives FOXC1-dependent myocardial senescence

**DOI:** 10.1093/nar/gkae274

**Published:** 2024-04-18

**Authors:** Bin Li, Weiyao Xiong, Wu Zuo, Yuanyuan Shi, Teng Wang, Lingling Chang, Yueheng Wu, Heng Ma, Qian Bian, Alex C Y Chang

**Affiliations:** Department of Cardiology and Shanghai Institute of Precision Medicine, Ninth People's Hospital, Shanghai Jiao Tong University School of Medicine, Shanghai 200125, China; Department of Cardiology and Shanghai Institute of Precision Medicine, Ninth People's Hospital, Shanghai Jiao Tong University School of Medicine, Shanghai 200125, China; Department of Cardiology and Shanghai Institute of Precision Medicine, Ninth People's Hospital, Shanghai Jiao Tong University School of Medicine, Shanghai 200125, China; Department of Cardiology and Shanghai Institute of Precision Medicine, Ninth People's Hospital, Shanghai Jiao Tong University School of Medicine, Shanghai 200125, China; Department of Cardiology and Shanghai Institute of Precision Medicine, Ninth People's Hospital, Shanghai Jiao Tong University School of Medicine, Shanghai 200125, China; Department of Cardiology and Shanghai Institute of Precision Medicine, Ninth People's Hospital, Shanghai Jiao Tong University School of Medicine, Shanghai 200125, China; Department of Cardiovascular Medicine, Guangdong General Hospital, Guangzhou, Guangdong, China; Department of Physiology and Pathophysiology, Fourth Military Medical University, No. 169 Changle West Rd, Xi’an 710032, China; Department of Cardiology and Shanghai Institute of Precision Medicine, Ninth People's Hospital, Shanghai Jiao Tong University School of Medicine, Shanghai 200125, China; Department of Cardiology and Shanghai Institute of Precision Medicine, Ninth People's Hospital, Shanghai Jiao Tong University School of Medicine, Shanghai 200125, China

## Abstract

Telomeres, TTAGGG_n_ DNA repeat sequences located at the ends of eukaryotic chromosomes, play a pivotal role in aging and are targets of DNA damage response. Although we and others have demonstrated presence of short telomeres in genetic cardiomyopathic and heart failure cardiomyocytes, little is known about the role of telomere lengths in cardiomyocyte. Here, we demonstrate that in heart failure patient cardiomyocytes, telomeres are shortened compared to healthy controls. We generated isogenic human induced pluripotent stem cell derived cardiomyocytes (hiPSC-CMs) with short telomeres (sTL-CMs) and normal telomeres (nTL-CMs) as model. Compared to nTL-CMs, short telomeres result in cardiac dysfunction and expression of senescent markers. Using Hi-C and RNASeq, we observe that short telomeres induced TAD insulation decrease near telomeric ends and this correlated with a transcription upregulation in sTL-CMs. FOXC1, a key transcription factor involved in early cardiogenesis, was upregulated in sTL-CMs and its protein levels were negatively correlated with telomere lengths in heart failure patients. Overexpression of FOXC1 induced hiPSC-CM aging, mitochondrial and contractile dysfunction; knockdown of FOXC1 rescued these phenotypes. Overall, the work presented demonstrate that increased chromatin accessibility due to telomere shortening resulted in the induction of FOXC1-dependent expression network responsible for contractile dysfunction and myocardial senescence.

## Graphical Abstract

**Graphical Abstract:**
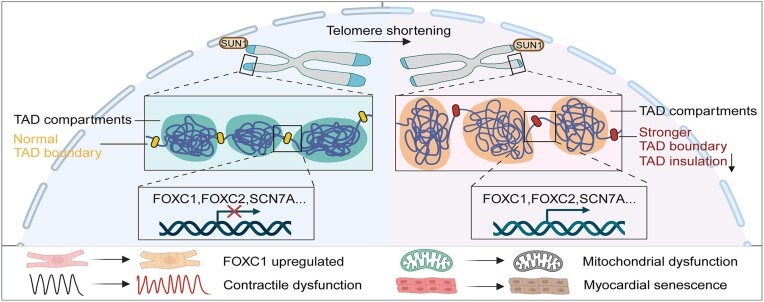

## Introduction

Aging contributes to the progression and severity of cardiac pathologies (1). As a major risk factor, aging increases the susceptibility of heart failure in older adults (2–4). Telomeres are tandem DNA repeats sequence (TTAGGG) bound by shelterin complex at chromosomal ends that function to preserve genomic integrity and stability (5–7). Due to replication end problem, telomeres shorten per cell cycle and critically short telomeres force cells into senescence thus defining the Hayflick limit (8). In stem cells and cancers, expression of telomerase serves to counteract telomere shortening therefore preserve regenerative capacity. Telomerase, comprised of telomerase reverse transcriptase (TERT) and an RNA template (telomerase RNA component, TERC) extends telomeric repeats in a cell cycle dependent manner (9,10). Critically short telomeres trigger persistent DNA damage response, induces cellular senescence and is considered as a hallmark of aging (11,12). Short telomeres have been implicated in pathologies associated with human aging, particularly in proliferative or regenerative organs (13–15). Besides acting as a gatekeeper for genomic stability, telomeres have also been implicated to participate in TAD formation (16), regulate transcription (17), and its transcription can also guide DNA repair or stabilize uncapped telomeric ends (18).

Despite mounting correlative evidence between short telomeres and cardiovascular diseases (1,12), whether telomeres participate in disease progression directly or indirectly remains unclear. Although less reported, we and others have demonstrated that in mouse (19,20), patient samples and human induced pluripotent stem cell derived cardiomyocytes (hiPSC-CMs), telomeres shorten independent of cell division (21–24). Propagation of TERT- or TERC-deficient mice to induce systemic short telomeres is sufficient to drive the onset of dilated cardiomyopathy in mice (25,26). We show that long telomeres can mask Duchenne cardiomyopathy (20) in mice and ‘pre-shortened’ telomeres causes early onset of heart failure with preserved ejection fraction when animals are challenged with hypertension/high fat diet (27). In patients, telomeres do shorten in cardiomyocytes of genetic and non-genetic cardiomyopathic patients independent of cell division and this phenomenon can be recapitulated in patient derived hiPSC-CMs (24,28). However, how shortened telomeres drive cardiac dysfunction independent of genetic mutations remains elusive.

Evidence demonstrating regulation of chromosomal three-dimensional (3D) structure and its ability to influence gene expression are emerging (29,30). Building on our understanding of transcription regulation and epigenetic regulation, it is now becoming more appreciated that distal regulatory elements also play an important role in regulating gene transcription during development, aging, and diseases (31,32). Using Hi-C (high-throughput/resolution chromosome conformation capture), active (A) and inactive (B) chromatin compartments and basic genome folding topologically associated domains (TADs) can be identified and studied. Although efforts have been invested to better understand how aging can regulate chromatin organization, person-to-person and animal-to-animal variability limits the conclusions.

To fill this gap in knowledge, we studied cardiac aging from the angle of shortened telomere lengths using an isogenic background system. We asked if shortened telomeres can regulate myocardial gene transcription in aged cardiomyocytes. By ‘pre-shortening’ telomere lengths, we confirm that short telomeres hiPSC-CMs (sTL-CMs) exhibit increased senescence markers, decreased mitochondrial respiratory reserve and contractile dysfunction compared to normal telomere length hiPSC-CM (nTL-CMs) controls. To identify key driver in myocardial senescence, we performed Hi-C and RNASeq analyses and identified upregulation of FOXC1 protein levels in sTL-CMs. The negative correlation between telomere length and FOXC1 was validated in heart biopsies (*n* = 26). Overexpression of FOXC1 in nTL-CMs and shFOXC1 knockdown in sTL-CMs experiments demonstrated causality, suggesting targeting telomeric homeostasis holds therapeutic potential despite cardiomyocytes’ non-dividing nature.

## Materials and methods

### Ethics

All studies using human iPSCs were reviewed and approved by the Ethics Review committee of Ninth People's Hospital, Shanghai Jiao Tong University School of Medicine (SH9H-2021-TK257-1). Human heart tissue samples were obtained from patients with end-stage heart failure (HF) that received heart transplantations or from biopsies of deidentified donor hearts used for heart transplantations in an Institutional Review Board–approved protocol at Guangdong General Hospital (Institutional Review Board No. GDREC2016255H). The collection and research use of these tissues was approved by the Ethics Committee of Guangdong General Hospital. Informed consent was obtained from all the participants.

### Cell culture, cardiac differentiation and generation of hiPSC-CMs with short telomeres

Human iPSCs were cultured on Matrigel (Corning) coated plates and grow in Nutristem hPSC XF medium (Biological Industries) at 37°C with daily media exchange. Cells were regularly checked for mycoplasma contamination. Cardiac differentiation was performed at a confluency of 70–90% using an established protocol (33). Briefly, to initiate differentiation, hiPSC were treated with 4–6 μM CHIR-99021 (Selleck Chemicals) for 2 days and followed by a Wnt inhibitor IWR-1 treatment (5 μM; Sigma) for another 2 days, RPMI 1640 medium (Sigma) supplemented with B27 minus insulin (Thermo Fisher Scientific) was used as the basal medium. Then, cells were refreshed in RPMI 1640 medium supplemented with B27 minus insulin. Two days later, the medium was switched to RPMI 1640 supplemented with B27 (Thermo Fisher Scientific) and maintained in this medium for 4 days with a medium change every other day. From day 11 of differentiation, beating hiPSC-CMs were purified using metabolic selection medium (RPMI 1640 without glucose, B27 supplement and 4 mM of Lactate) and changed every two days. For the generation of short telomere hiPSC-CMs, hiPSCs (UC 3–4) (34) pre-treated with BIBR1532 (20 μM) at the pluripotent stage were proceeded with cardiomyocyte differentiation and maintained treatment during the differentiation process until day 11 of differentiation. For hiPSC-CMs, the bilogical replicates were defined as different rounds of differentiation.

### RNA isolation and real time-quantitative PCR (RT-qPCR)

Total RNA was extracted using TRIzol (Invitrogen) according to the manufacturer's instructions. 100 ng RNA was used to generate cDNA by using the HiScript® II 1st Strand cDNA Synthesis Kit (Vazyme). RT-qPCR was performed on LightCycler @ 480 II (Roche) using a ChamQ Universal SYBR qPCR Master Mix (Vazyme). The relative mRNA expression levels were calculated by the 2^−ΔΔCt^ method and normalized to GAPDH mRNA. Sequences of primers used are listed in Supplementary Table 2.

### Absolute average telomere length detection

Genomic DNA was extracted from cell pellets using EasyPure Blood Genomic DNA Kit (TransGen Biotech) and quantified by measuring OD_260_. DNA quality control criteria were determined using the OD_260_/OD_280_ ratio between 1.8 and 2.0. Absolute average telomere length was measured using Absolute Human Telomere Length Quantification qPCR Assay Kit (ScienCell) according to the manufacturer's instructions.

### Immunofluorescence and Immunohistochemical Staining

Cells were grown on sterile TC coverslips (Fisherbrand), washed with PBS and fixed in 4% formaldehyde for 10 min at room temperature. After three PBS washes, cells were permeabilized with staining buffer (0.1% Triton X-100 and 20% FBS in PBS, all from Sigma). Cells were then incubated in primary antibody overnight at 4°C followed by incubation in secondary antibody for 1h at room temperature. Primary antibodies used: Rabbit monoclonal anti-phospho-histone H_2_AX (Ser 139) (1:1000 dilution, 9718S, Cell Signaling Technology); p21 Waf1/Cip1 (12D1) Rabbit mAb (1:500 dilution, 2947S, Cell Signaling Technology); Mouse monoclonal anti-cardiac troponinT (1:400 dilution, ab8295, Abcam); FOXC1 (D8A6) Rabbit mAb (1:200 dilution, 8758S, Cell Signaling Technology); SUN1(1:1000) (35); SCN7A antibody (1:200 dilution, NB100-81029, Novus); ATL3 Polyclonal Antibody (1:200 dilution, PA5-98495, Invitrogen); ENO1 Polyclonal antibody (1:100 dilution, 11204-1-AP, Proteintech). Secondary antibodies used: Alexa Fluor 488, Alexa Fluor594 or Alexa Fluor647 (1:1000 dilution, Invitrogen). After washed three times with staining buffer, samples were incubated with PBS containing 4,6-diamidino-2-phenylindole (DAPI) for 10 min and mounted with VECTASHIELD^®^ Mounting Medium (Vectorlabs). For image acquisition, a Zeiss LSM880 confocal microscope was used.

For immunofluorescence staining of human cardiac microtissues, primary antibodies used: Mouse monoclonal anti-cardiac troponinT (1:400 dilution, ab8295, Abcam); Anti-Vimentin antibody (1:1000 dilution, ab92547, Abcam); Human CD31/PECAM-1 Antibody (1:400 dilution, AF806, R&D Systems). For immunohistochemistry of the human heart biopsies, tissues were fixed overnight in 4% paraformaldehyde and embedded, sections were deparaffinized and rehydrated. Then, antigen-retrieved sections were incubated in 0.3% hydrogen peroxide and incubated with primary antibodies (Goat polyclonal to FOXC1: 1:200 dilution, NB100-1268, Novus; Goat polyclonal to FOXC2: 1:200 dilution, ab5060, Abcam) at 4°C overnight followed by secondary antibodies incubation.

### Telomere Q-FISH, immuno-FISH and image acquisition

Cell samples were fixed with 4% paraformaldehyde in PBS solution for 5 min at room temperature and subsequently maintained in PBS solution at 4°C. For hiPSC-CMs, cells were replated on day 27 of differentiation onto Matrigel-coated eight-chamber slides and fixed after 3 days. Paraffin-embedded cardiac tissue sections were deparaffinized in xylene (Sigma) and dehydrated in an ethanol series (70%, 90% and 100%; 5 min each). Telomere Q-FISH was performed as previously described by using TelC-Cy3 PNA probe (CCCTAACCCTAACCCTAA, F1002, PNA Bio) (24). For immuno-FISH, tissues or cells were further blocked with staining buffer (20% FBS/0.1%Triton X-100/PBS solution) and stained with prediluted primary antibody: anti-cardiac troponinT (1:500 dilution, ab8295, Abcam) or anti -phospho-histone H_2_AX (Ser 139) (1:500 dilution, 9718S, Cell Signaling Technology), 53BP1 (1:500 dilution, IHC-00001, Bethyl) for 2 h at room temperature in staining buffer. After washed with PBS solution, samples were incubated with secondary antibodies for 1h and counterstained with DAPI (4 μg / ml) in PBS solution for 5 min, washed with distilled H_2_O, air-dried, and mounted with VECTASHIELD^®^ Mounting Medium (Vectorlabs). For cells, confocal images were acquired as stacks for a total of 4 μm, while tissue confocal images were acquired as stacks every 1 μm for a total of 5 μm using a Zeiss LSM880 confocal microscope at 60× objective, and maximum projections were done with Zen software. Telomere length was determined by PNA signal intensity and quantified using Imaris (Bitplane).

### Detection of doxorubicin vulnerability in hiPSC-CMs

For IC50 detection, given that hiPSC-CMs do not proliferate, we selected TUNEL (Elabscience) staining to detect IC50 of doxorubicin. nTL-CMs and sTL-CMs were challenged with different concentration of doxorubicin (10^−3^–10^2^ μM, Sigma-Aldrich) for 48h and TUNEL staining was performed per manufacturer's instructions. Micrographs were captured using an Olympus IX83 microscopy. A minimum of 10 fields of view and total 180–250 cells were analyzed for each concentration. Proportion of TUNEL positive cells in each doxorubicin concentrations were used to calculate the IC50 using Graphpad Prism 9.

### Senescence-associated β-galactosidase (SA-β-Gal) activity

SA-β-Gal activity was assessed using a senescence associated β-galactosidase staining kit (Solarbio). In brief, cells were fixed with fixation buffer containing formaldehyde for 5 min. Next, the cells were treated with staining buffer containing X-gal overnight at 37°C. Stained cells were washed using PBS and observed by Olympus IX83 microscopy.

### RNA-sequencing

RNA-sequencing was performed by Novogene Bioinformatics Technology using the Illumina HiSeq instrument. Total RNA from day 30 nTL- and sTL-hiPSC-CMs were isolated using the TRIzol Reagent (Life Technologies). Amounts and integrity of total RNA were assessed using the RNA Nano 6000 Assay Kit for Bioanalyzer 2100 system (Agilent Technologies). Differential expression analysis was performed using the DESeq2 R package (1.20.0). Benjamini and Hochberg's approach were used to adjust the resulting *P*-values for controlling the false discovery rate. |log_2_(foldchange)| > 1 and *P*-adj < 0.05 were set as the threshold.

### Hi-C library construction

Hi-C libraries for normal and short telomere hiPSC-CM were constructed with previously described *in situ* Hi-C protocols with minor modifications (36). Cells were fixed with 0.92 ml of 1% v/v formaldehyde at RT for 10 min. Next, 0.08 ml of 2.5 M glycine was added. The samples were incubated at RT for 5 min and on ice for at least 15 min to quench crosslinking completely. The fixed samples were collected by centrifuging and then lysed with 1 ml of ice-cold lysis buffer (10 mM Tris–HCl pH 8.0, 10 mM NaCl, 0.2% Igepal CA630) with protease inhibitors subsequently. The samples were incubated on ice for 30 min and centrifuged for 5 min at 3000 × g at 4°C to collect the nuclei. The nuclear pellets were washed once with NEBuffer 3.1, then resuspended in 20 μl of 10 × NEBuffer 3.1, 140 μl of ddH2O, and 40 μl of 0.5% SDS; and then incubated at 65°C for 5 min to open up chromatin. Next, 22 μl of 10% Triton X-100 (Sigma) was added before incubation at 37°C for 15 min to quench SDS. 5 μl of 10 × NEBuffer 3.1, 15 μl of ddH2O, and 8 μl (400 U) of *Dpn*II (NEB) were added to the samples to digest chromatin. Digestion was performed overnight at 37°C on a thermomixer with interval shaking. The samples were incubated at 65°C for 20 min to inactivate the restriction enzyme. To fill the restriction overhangs, 60 μl of fill-in mix (37.5 μl of 0.4 mM biotin-14-dATP (Life Technologies), 1.5 μl of 10 mM dCTP/dGTP/dTTP, 10 μl of 5 U/μl Klenow, 6 μl of 10 × NEBuffer 3.1, and 2 μl of Milli-Q water) was added to the reaction, and the samples were incubated at 25°C for 4 h. Ligation mix (498 μl of water, 240 μl of 5 × T4 DNA ligase buffer (Invitrogen), 100 μl of 10% Triton X-100, 12 μl of 10 mg/ml BSA and 50 μl of 1 U/μl T4 DNA ligase (Invitrogen) were then added into the samples. The samples were incubated at 16°C for exactly 4 h. After ligation, the samples were centrifuged and then resuspended in 500 μl of 1 × NEBuffer. To reverse crosslinking, 30 μl of 20 mg/ml proteinase K (NEB) and 50 μl of 10% SDS (Sigma) were added to the samples, and the samples were then incubated at 65°C overnight. Phenol–chloroform extraction and ethanol precipitation were performed. The purified DNA was sheared with a Covaris M220. End repair of DNA fragments and ligation with adapters were performed using the End Repair/dA-Tailing Module (NEB) and the Ligation Module (NEB) according to the product manuals after DNA shearing. To purify biotinylated DNA fragments, Dynabeads MyOne Streptavidin T1 beads (Life Technologies) were used to bind the biotinylated DNA. The beads were washed twice with 500 μl of 1 × TWB on a Thermomixer at 55°C for 2 min with mixing and resuspended in 50 μl of Milli-Q water. The samples were amplified with Phusion High-Fidelity DNA Polymerase (Thermo Fisher) for 6–8 cycles. AMPure XP beads (Beckman Coulter) were agopted for size selection. All Hi-C libraries were sequenced on an Illumina NovaSeq 6000 platform in PE150 mode.

### Hi-C data processing

#### Mapping, binning, evaluation and Heatmap generation

The Hi-C reads were mapped to the hg38 genome and filtered using the hiclib pipeline (https://bitbucket.org/mirnylab/hiclib) as reported previously (37). The mapped Hi-C fragments of replicates were combined and converted into cool format using the cooler package (https://github.com/mirnylab/cooler) (38). The cool files were balanced and binned into multiple resolutions (10 kb, 50 kb, 500 kb and 1 Mb) for subsequent downstream processing. The *cis* and trans expected contact probability at different genomic separations was calculated using the cooltools package (https://github.com/mirnylab/cooltools) (39). The observed heatmaps and observed/expected heatmaps at 10 kb, 50 kb and 1 Mb resolutions in the figures were generated using scripts derived from the cooltools package.

#### Evaluation of reproducibility of Hi-C replicates

Pearson correlation coefficients between Hi-C replicates for each stage or between datasets of different stages were calculated using cool files binned at 500 kb and the HiCRep package (40). The optimal smoothing parameter was set as 1, which was determined by the function htrain, and the lower and upper bounds of the genomic distance between interaction loci were set to 0 and 5000 000 as recommended. Reproducibility scores for the whole genome of each sample were generated as the mean value of all chromosomes.

#### *P(s)* curves and loop size derivation

Contact probability (*P*[*s*]) curves were computed from the cool files binned at 10 kb resolution. The linear genomic separations were divided into logarithmic bins with a factor of 1.12. The *P*(*s*) values were calculated by averaging the chromatin interaction frequencies within each log-spaced bin for the combined autosomes as well as individual autosomes. The *P*(*s*) values were plotted in log space versus the genomic distances using custom R scripts to generate *P*(*s*) curves.

### TAD (topologically associated domain) analysis

Insulation analysis and TAD calling were performed on cooler matrices of combined Hi-C replicates binned at 10 kb resolution using the diamond-insulation utility from cooltools. The insulation score for each 10 kb genomic bin was calculated using an ‘insulation square’ approach as described previously (37). Briefly, a 500 kb × 500 kb square (50 bins × 50 bins) with the bottom-right corner touching the diagonal of the Hi-C interaction matrix was slid along the diagonal. For each 10 kb genomic bin, the interactions within the square were aggregated, representing the total interactions occurring across the bin. To generate the insulation score, the aggregated interaction score at each position was log_2_ normalized to the mean interaction score across the entire chromosome. We generated cumulative frequency curves for the insulation scores in the telomere-proximal and telomere-distal regions and compared the differences in insulation scores between nTL-CMs and sTL-CMs by performing a paired Wilcoxon rank sum test. We further calculated the differences in insulation scores (insdiff) by subtracting the insulation score in nTL-CMs from that in sTL-CMs for each 10 kb genomic bin. Comparison between the insulation differences for the telomere-proximal and telomere-distal regions was again performed using the Wilcoxon rank sum test. TAD boundaries were identified using the default parameter. A noise threshold of 0.1 was further used to select the most prominent TAD boundaries. Insulation profiles were plotted using custom scripts in R.

### Lentiviral transduction of hiPSC-CMs

For gene overexpression, FOXC1 cDNA was PCR amplified and cloned into the target gene lentiviral plasmid (pLVX-ZsGreen1 backbone). HEK293T cells were transfected with packaging plasmids using Lipofectamine 2000 according to the manufacturer's protocol (Life Technologies). After 24 h, supernatant of the transfected cells was collected and centrifuged at 6000 g for 15 min, then, concentrated using Lenti-X Concentrator (Clontech). HiPSC-CMs were infected with the lentivirus for 24 h.

### IMP and EFP measurements

Impedance (IMP) and extracellular field potential (EFP) were detected using CardioExcyte 96 (Nanion Technologies) according to the standard procedures. Briefly, cardiomyocytes were seeded on CardioExcyte 96 Sensor Plates (pre-coated overnight with Matrigel, Nanion Technologies) at 5 × 10^4^ cells per well. On the day of measurement, fresh medium was changed 2 h prior to assay. Electrophysiology of spontaneous beating hiPSC-CMs was measured using the CardioExcyte Control software and analyzed using DataControl 96 software (Nanion Technologies).

### Mitochondrial respiration measurement

For mitochondrial respiration measurements, 1 × 10^5^ hiPSC-CMs were seeded onto matrigel-coated XFe96 Microplates (Agilent) per well for 3 days. Culture medium was replaced with XF RPMI Base Medium supplemented with 5 μmol/L glucose, 1 μmol/l pyruvate, and 10 μmol/l glutamine (all from Agilent), and transferred into a CO_2_-free incubator for 1 h at 37°C. Then, baseline oxygen consumption rate (OCR) was measured, followed by oligomycin, oxidative phosphorylation uncoupler FCCP, rotenone and antimycin A injection as manufacturer's instructions.

### CUT&Tag library generation and sequencing

CUT&Tag assay was performed using Hyperactive Universal CUT&Tag Assay Kit for Illumina Pro (Vazyme) as indicated. Briefly, 1 × 10^5^ cells were treated with ConA beads, then incubated with primary antibody (Goat polyclonal to FOXC1: 1:50 dilution, NB100-1268, Novus; Rabbit polyclonal to Histone H3 (acetyl K27): 1:50 dilution, ab4729, Abcam; Tri-Methyl-Histone H3 (Lys27) (C36B11) Rabbit mAb: 1:50 dilution, 9733S, Cell Signaling Technology) overnight at 4°C. Cells were reacted with secondary antibody for 1 h after being washed for three times. Subsequently, samples were incubated with pAG-Tn5 for 1 h. DNA tagmentation was performed at 37°C for 1 h and followed by DNA extraction. PCR was applied to amplify the libraries followed by product purification. Sequencing and data analysis were performed by Novogene Co. Ltd using Novaseq-PE150 (Illumina).

## 3D cardiac microtissues (MTs) culture and contractility assay

Cardiac MTs were generated using 3D Petri Dish from MicroTissues Inc per manufacturer's instructions. Briefly, 3D Petri Dish micro-molds were casted and carefully transferred to new tissue culture dish. Cell suspensions were prepared according to the proportions previously described with minor modifications (41), which combined of a total of 3000 cells (70% cardiomyocytes (nTL-CMs or sTL-CMs), 15% endothelial cells and 15% fibroblasts). Cell suspensions were mixed and cultured in B27 medium supplemented with VEGF (50 ng/ml) and FGF2 (5 ng/ml) and seeded onto the 3D Petri Dish to form MT clusters. MTs were incubated at 37°C, 5% CO_2_ for 21 days with media refreshed every 3–4 days.

Day21 cardiac MTs with varying proportions of sTL-CMs were seeded on 24-well plates prior to assay. Videos of spontaneous beating cardiac MTs were acquired using an Olympus IX83 microscopy for at least 10 s at 37°C and 5% CO_2_. Bright-field videos were captured at 60 fps and a 10× phase contrast objective using an Olympus IX83 microscopy. At least 15 separated cardiac MTs were used per experimental condition. Contraction data were obtained by analyzing movies with an established Conklin method algorithm. From the contraction/relaxation profile, velocity and the beating rate were analyzed over at least 10 repetitive contractions. Quantification was calculated using Matlab-based motion-tracking software as previously described (42).

### Statistical analysis

All experimental data are presented as mean ± SEM. Statistical significance between two groups was determined using two-tailed Student's *t*-test. Differences between more than two groups were compared using One-way ANOVA with Tukey's multiple comparisons test. Data were analyzed and represented with GraphPad Prism version 8.01 (GraphPad Software, Inc.). Statistical significance was considered at *P< 0.05, **P< 0.01 and ***P< 0.001.

## Results

### Short telomere cardiomyocytes are senescent and dysfunctional

**Figure 1.**
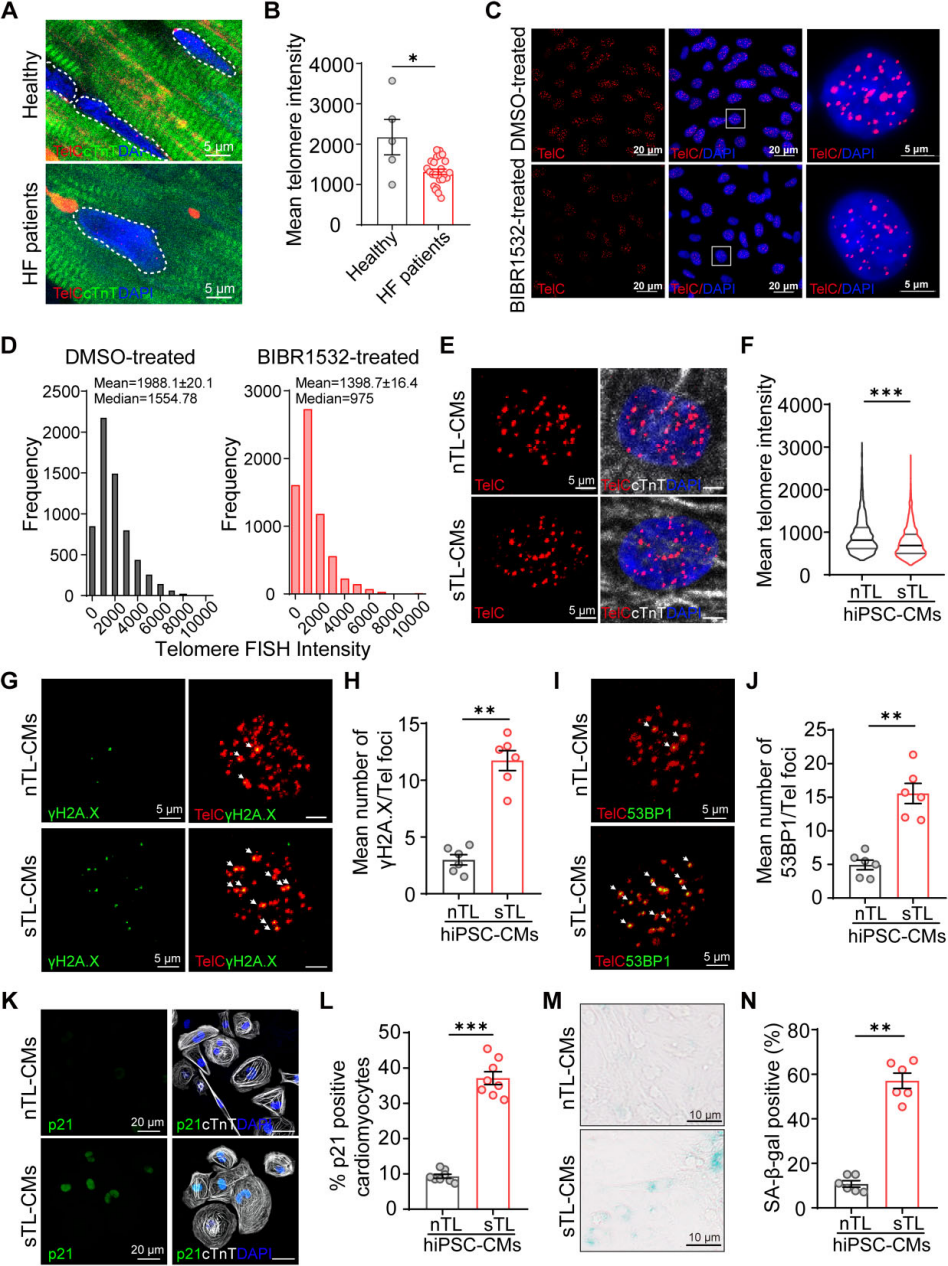
Shortened telomeres induced hiPSC-CMs senescence. (**A**) Representative Q-FISH micrographs and (**B**) quantification of mean myocardial telomere lengths of healthy (*n* = 5) and heart failure (HF) patients (*n* = 24). (**C**) Representative Q-FISH micrographs of day 24 normal telomere (DMSO-treated) and short telomere hiPSCs (BIBR1532-treated). Telomeres were visualized with TelC probe (red). High magnification micrographs of selected nuclei are shown (DMSO-treated hiPSCs, *n* = 132 cells; BIBR1532-treated hiPSCs, *n* = 126 cells, biological, *n* = 3). (**D**) Histograms displaying telomere intensity distribution in DMSO- and BIBR1532-treated hiPSCs. (**E, F**) Representative Q-FISH micrographs and quantification of mean telomere intensity of nTL- and sTL-CMs (nTL-CMs: *n* = 82; sTL-CMs: *n* = 102, biological, *n* = 3). (**G**) Representative micrographs of hiPSC-CMs co-stained for DNA damage γH_2_A.X and telomeres. White arrows indicate co-localization of γH_2_A.X at telomeric ends. (**H**) Mean number of telomeric γH_2_A.X foci (biological, *n* = 6). (**I, J)** Representative micrographs and quantification of hiPSC-CMs co-stained for 53BP1 and telomeres (biological, *n* = 6). (**K, L**) Representative micrographs and quantification of p21 expression in nTL- and sTL-CMs (biological, *n* = 8). (**M, N**) Representative micrographs and quantification of SA-β-gal activity in nTL- and sTL-CMs (biological, *n* = 6). **P≤ 0.01 and ***P≤ 0.001. Data are presented as mean values ± SEM.

Previously we showed that diseased cardiomyocytes due to structural deficiencies exhibit telomere shortening (20,24) and in non-genetic heart failure patients we also detect telomere length shortening (Figure 1A and B). To delineate the contribution of short telomeres apart from structure deficiencies in myocardial dysfunction, hiPSCs were cultured in presence of telomerase inhibitor (2-[(*E*)-3-naphthalen-2-yl-but-2-enoylamino]-benzoic acid; BIBR1532) to generate isogenic normal and short telomere hiPSCs which were subsequently differentiated into beating cardiomyocytes (43). hiPSCs were treated with either BIBR1532 or DMSO (control) for 24 days and relative telomere lengths were quantified using Q-FISH as previously described (24). BIBR1532 treatment resulted in a decrease (DMSO-treated: 1988.1 ± 20.1; BIBR1532-treated: 1398.7 ± 16.4) in mean telomere signal in hiPSCs compared to DMSO controls (Figure 1C and D). We further measured the absolute mean telomere length of hiPSC using standardized RT-qPCR method. DMSO treated hiPSCs exhibit 17.6 ± 0.3 kb telomeric repeats per chromosome end (defined as normal telomere length, nTL) while BIBR treatment resulted in ∼30% telomere loss (12.5 ± 0.5 kb telomeric repeats per chromosome end) and is defined as short (sTL) (Supplementary Figure S1A). sTL-hiPSCs maintained pluripotency marked by OCT4, SOX2 expression and the ability to generate all three germ layers in teratoma assay (Supplementary Figure S1B-D). Further, short telomeres had no effect in hiPSCs survival and no karyotypic abnormalities were observed in sTL-hiPSCs (Supplementary Figure S1E, F). To test whether telomere length loss is reversible, we further cultured sTL-hiPSCs for another 24 days in absence of BIBR1532 and measured telomere levels. BIBR1532 removal allowed telomere length increase in sTL-hiPSCs (Supplementary Figure S1G and H). These results provide us the necessary cell model to investigate role of telomeres in cardiomyocytes in an isogenic manner without affecting pluripotency of hiPSCs.

Next, we proceeded with cardiomyocyte differentiation. During differentiation, we did not detect any differences in cardiac lineage commitment between nTL- and sTL-hiPSCs (Supplementary Figure S2). We confirmed that sTL-CMs exhibited significantly shortened telomeres (Figure 1E and F). Among the telomere-binding shelterin genes, only POT1 and TPP1 were significantly decreased in sTL-CMs compared to nTL-CMs (Supplementary Figure S3). Telomeric dysfunction and DNA damage response marked by γH_2_A.X and 53BP1 (Figure 1G, J, Supplementary Figure S4A–D) were significantly increased in sTL-CMs compared to nTL-CMs. Next, we asked if sTL-CMs enter into senescence like murine cardiomyocytes (20). Compared to nTL-CMs, sTL-CMs exhibited a significant upregulation of p21 (Figure 1K and L) and p16 proteins (Supplementary Figure S4E and F), increased cell size (Supplementary Figure S4G), as well as increased SA-β-Gal activity (Figure 1M and N).

**Figure 2.**
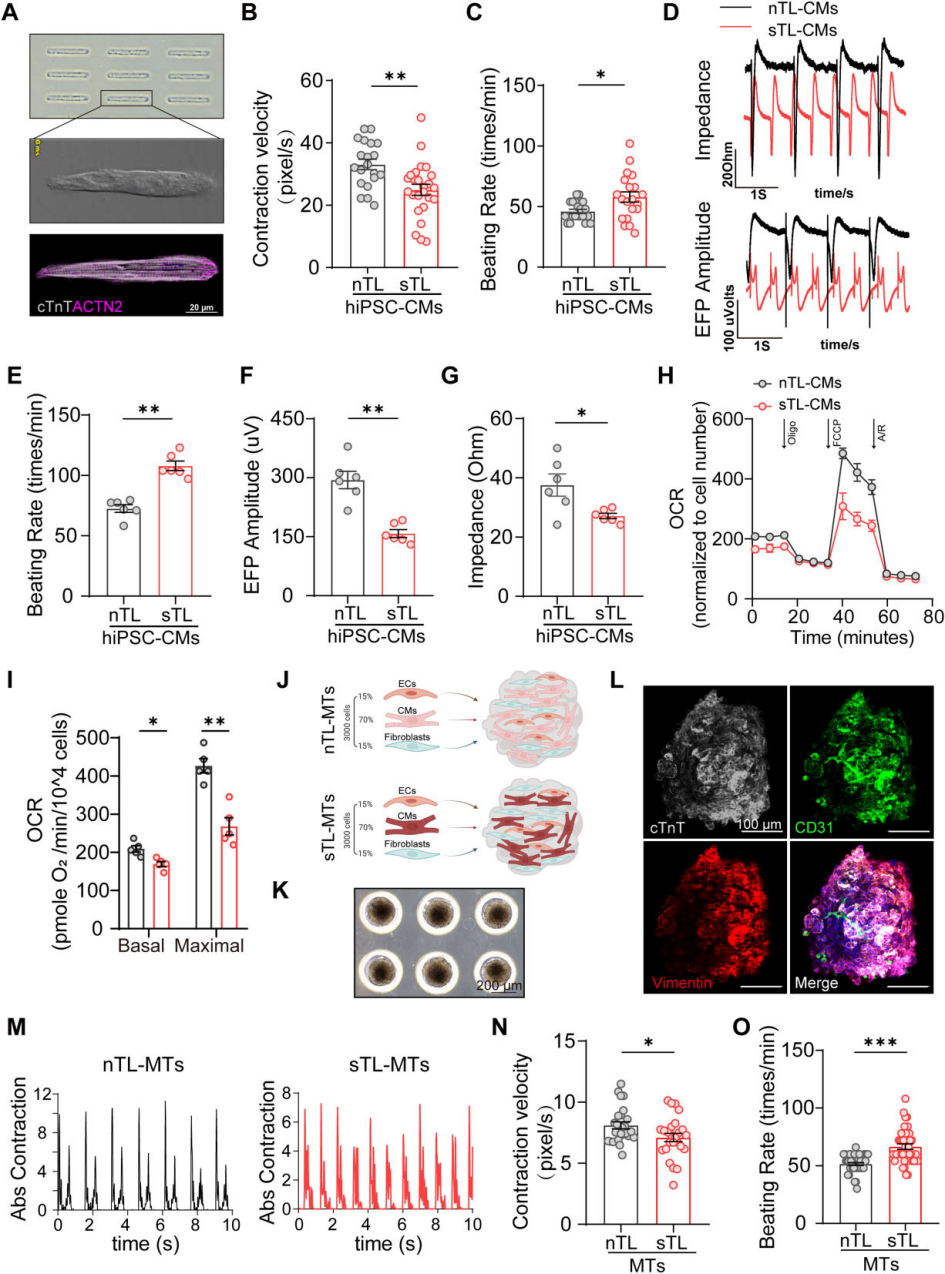
STL-CMs exhibit contractile dysfunction. (**A**) Micropatterning of hiPSC-CMs to promote sarcomeric alignment. (**B**) Contraction velocity and, (**C**) beating rate of micropatterned hiPSC-CMs (*n* = 20 cells for each group, biological, *n* = 2). (**D**) Representative impedance (IMP) and extracellular field potential (EFP) traces of nTL- and sTL-CM monolayers are shown. (**E–G**) sTL-CMs exhibit aberrant beating rates, EFP and IMP compared to nTL-CMs (biological, *n* = 6). (**H**) Real-time mitochondrial respiration (oxygen consumption rate, OCR) of nTL- and sTL-CMs were measured and (**I**) basal and maximal OCR values are shown (biological, *n* = 5). (**J**) Schematic showing generation of nTL-MTs and sTL-MTs. (**K, L**) Bright field image and representative immunofluorescence micrographs of MTs stained for cardiomyocytes (cTnT, gray), endothelial cells (CD31, green) and fibroblast (vimentin, red). (**M**) Representative motion traces of nTL- and sTL-MTs. (**N**) Contraction velocity and (**O**) beating rate are shown. (n > 15 MTs per group, biological, *n* = 2). *P≤ 0.05, **P≤ 0.01 and ***P≤ 0.001. Data are presented as mean values ± SEM.

Functionally, we evaluated cardiomyocyte contraction on single (Figure 2A), monolayer and 3D cultured microtissues (MTs) (41) as well as mitochondrial respiration. In keep with prior animal observations (26), sTL-CMs exhibited decreased contraction velocity and increased beating frequency at single (Figure 2B and C, Supplementary Figure S4H) and monolayer (Figure 2D-G) culture conditions. Further, sTL-CMs exhibited lower basal and maximal mitochondrial respiratory capacity compared to nTL-CMs (Figure 2H and I). To generate 3D microtissues, nTL- or sTL-CMs (cardiac troponin T; cTnT) were mixed with endothelial (CD31) and fibroblast cells (Vimentin) and cultured for three weeks before contraction function was evaluated (Figure 2J-L). Like monolayer cultures, sTL-CM MTs exhibit elevated p21 levels (Supplementary Figure S4I and J). Functionally, presence of sTL-CMs resulted in decreased contraction velocity (Figure 2M and N) and increased beating frequency (Figure 2O) in MTs. Finally, to evaluate whether sTL-CMs exhibit more susceptibility to doxorubicin challenge than nTL-CMs, we challenged the hiPSC-CMs with doxorubicin (DOX) as a model of DOX-induced cardiotoxicity (44). Compared to nTL-CMs (IC50 = 8.36 μM), sTL-CMs (IC50 = 1.84 μM) showed reduced tolerance to doxorubicin after a 48h treatment (Supplementary Figure S4K). Further, greater doxorubicin cardiotoxicity was observed in sTL-MTs compared to nTL-MTs reflected in a decrease in contraction velocity (Supplementary Figure S4L). Together, these results demonstrate that short telomeres, in absence of other genetic variations, is capable of inducing senescence-like state in hiPSC-CMs. Functionally, these ‘aged’ CMs exhibit mitochondrial, contractile dysfunction and are more vulnerable to DOX-challenge.

### Short telomeres induce chromatin decompaction near telomeric regions

**Figure 3.**
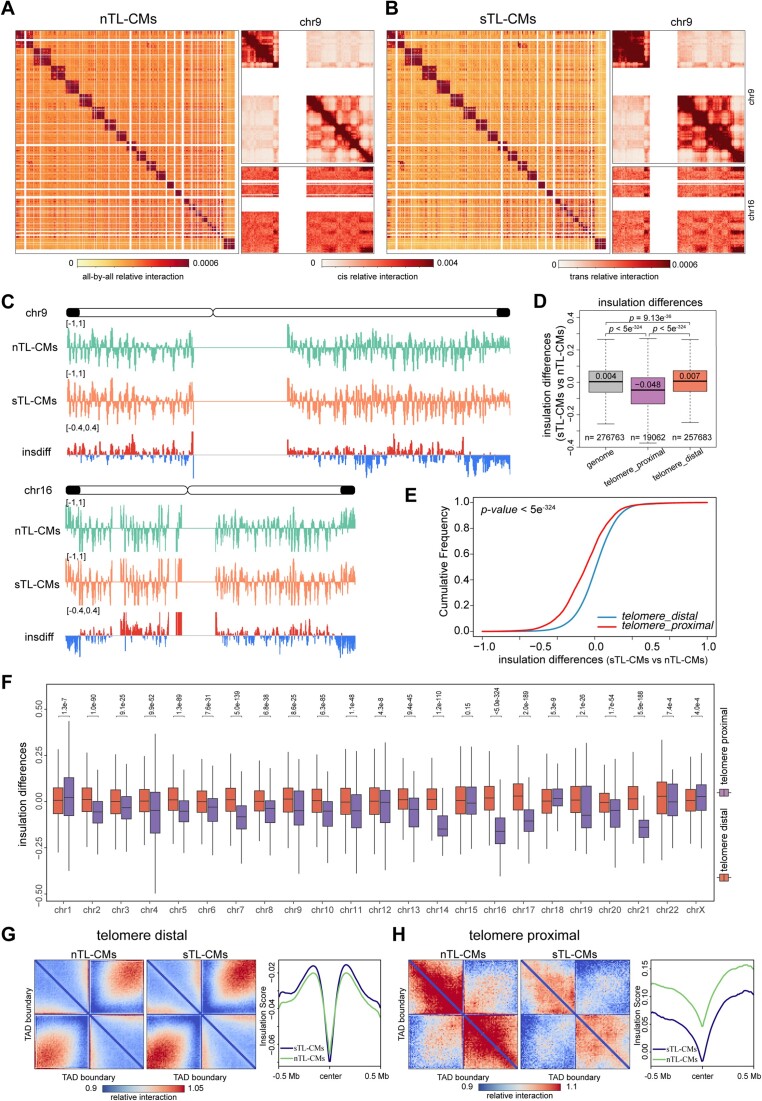
sTL-CMs exhibit pronounced chromosomal decompaction at telomere proximal regions. (**A, B**) Genome-wide Hi-C interaction heatmaps of nTL- and sTL-CMs 3D genome architecture binned at 1 Mb resolution show reorganization of 3D genome architecture due to short telomeres. Enlarged Chr9 and Chr9-Chr16 *trans* interaction heatmaps are shown at right. (**C**) Representative images showing insulation differences between nTL- and sTL-CMs. Blue parts indicate decreased insulation score in sTL-CMs compared to nTL-CMs. (**D**) Insulation differences of whole genome (all) and chromatin within/over 5 Mb from telomeric ends between sTL- and nTL-CMs. grey box: whole genome; purple box: within 5 Mb from telomeric ends; orange box: over 5 Mb from telomeric ends. (**E**) Cumulative frequency plots of insulation difference in telomere-proximal (within 5 Mb) versus telomere-distal (over 5 Mb) regions. (**F**) Insulation differences of all chromosomes within/over 5 Mb from telomeric ends between nTL- and sTL-CMs. (**G, H**) Pileups of interactions between TAD boundaries distal and proximal to the telomeric end and their surrounding regions within 500 kb. Bin size, 10 kb. Averaged insulation profiles for 1 Mb genomic regions centered at TAD boundary distal/proximal to the telomeric ends are shown.

To assess how shortened telomeres may alter chromatin conformation and gene expression in cardiomyocytes, high-throughput chromosome conformation capture (Hi-C) was performed in two biological replicates of nTL- and sTL-CMs. Pearson correlation coefficients between the biological replicates were calculated using HiCRep to confirm the reproducibility of our Hi-C datasets (Supplementary Figure S5A). The overall heatmap (Figure 3A and B) and P(s) curve (Supplementary Figure S5B) indicating chromatin conformation altered due to shortened telomeres in hiPSC-CMs. The inter-chromosomal interactions were significantly decreased in sTL-CMs compared to nTL-CMs (Figure 3A and B, Supplementary Figure S5C, D, Supplementary Table S1). A majority of transcriptionally active A compartment and inactive B compartment regions showed moderate alteration (Supplementary Figure S5E and F).

We further calculated the insulation score by aggregating the chromatin interactions across each 10 kb genomic bin and generated the insulation difference between nTL- and sTL-CMs. Intriguingly, the insulation scores significantly decreased near telomeric regions in sTL-CMs (Figure 3C), indicating that the telomere-proximal regions adopt a more extended conformation in sTL-CMs. We next quantitively compared the insulation differences for genomic bins within telomere-proximal regions versus those within the telomere-distal regions. Insulation scores decreased significantly within telomere-proximal regions both 5 Mb and 15 Mb proximal to telomeric ends in sTL-CMs compared to nTL-CMs (Figure 3D and Supplementary Figure S5G). To further highlight the differences in chromatin structure changes in telomere-proximal versus telomere-distal regions, we computed the insulation difference for each 10 kb bin (sTL-CMs minus nTL-CMs). When plotting the insulation differences as cumulative frequency plots, it is evident that the insulation differences for the telomere proximal bins are more negative than those for the telomere distal bins (Figure 3E and Supplementary Figure S5H), suggesting the telomere proximal bins exhibit greater decreases in insulation scores upon telomere shortening than the telomere distal bins. We further assessed the insulation differences at telomere proximal regions (5 Mb) of individual chromosome and noticed that the most pronounced insulation decreases observed at chr9, chr16, chr19, and chr21 (Figure 3F). The varying degrees of changes in insulation scores on different chromosomes are not correlated with chromosome lengths, chromosome arm lengths (Supplementary Figure S6) or whether the chromosomes are acrocentric (Supplementary Figure S7). Collectively, these analyses suggest that telomere shortening could induce chromatin conformation changes at chromosomal ends.

As the local minima of the insulation scores denote TAD (topologically associated domain) boundaries, we assessed whether the insulation decrease at telomeric ends influences TAD organization. We generated Hi-C interaction pileups for 1 Mb genomic windows centered at TAD boundaries distal/proximal to telomeric ends (Figure 3G and H, Supplementary Figure S8A-E). At telomere distal regions, the intra-TAD and cross-boundary interactions remain largely unchanged in sTL-CMs compared to nTL-CMs (Figure 3G, left). Concordantly, the averaged insulation profiles for telomere-distal TAD boundaries are nearly identical (Figure 3G, right). Interestingly, for TADs within telomere-proximal regions, both the intra-TAD and cross-boundary interactions decreased in sTL-CMs (Figure 3H, left), while the averaged insulation profiles exhibited a downward shift (Figure 3H, right). Thus, the shortening of telomeres in sTL-CMs did not cause a significant reduction in TAD boundary strengths. Rather, it resulted in a uniform loss of chromatin interactions near the telomeric ends. Taken together, our results indicate that hiPSC-CMs harboring short telomeres exhibit pronounced chromosomal decompaction at telomere proximal regions.

We asked if these higher-order chromatin structural changes were linked to changes in epigenetic marks. To this end, we performed H3K27ac and H3K27me3 CUT&Tag experiments on nTL-CMs and sTL-CMs. Both H3K27ac and H3K27me3 marks were increased in sTL-CMs compared to nTL-CMs (Supplementary Figure S9A). We next compared the extent of changes of H3K27Ac and H3K27me3 signals in telomere-proximal versus telomere-distal regions (Supplementary Figure S9B–E). On 17 of 23 chromosomes, the telomere-proximal regions exhibit greater increases in H3K27ac signals than the telomere-distal regions (Supplementary Figure S9B, D), indicative of a more open chromatin state. Interestingly, H3K27me3, which can be both an epigenetic repressive mark but also a TERRA (telomeric repeat-containing RNA) guided telomeric histone mark (45), was also elevated in telomere-proximal regions in sTL-CMs compared to nTL-CMs (Supplementary Figure S9C, E). It has been shown that short telomeres can increased TERRA levels (46,47). To evaluate this, we measured TERRA levels by RT-qPCR using published chromosome-specific primers (48). Indeed, sTL-CMs exhibit an increase in TERRA expression on different chromosomes compared to nTL-CMs (Supplementary Figure S10 and Supplementary Table S3). Thus, the increase in H3K27me3 signals in telomere-proximal regions might be a result of both short telomeres and TERRA-dependent epigenetic regulation, in line with previous report (49).

We further evaluated the correlation between the changes of higher-order chromatin structure measured by Hi-C and epigenetic marks. We calculate the insulation differences and the fold changes of H3K27ac and H3K27me3 signals in distal (mid) and telomere-proximal regions at p-arms (p-proximal) and q-arm (q-proximal) for all chromosomes (Supplementary Figure S11A-D). Notably, the insulation differences negatively correlate with the changes in H3K27ac or H3K27me3 for both the ends of p- (p-proximal) and q-arms (q-proximal) (Supplementary Figure S11E and F). In other words, the chromosome ends exhibiting greater increases in H3K27ac and H3K27me3 also exhibit more substantial decreases in insulation scores, indicative greater chromatin decompaction. Taken together, our data suggest that the telomere shortening affects the distribution of epigenetic marks and the chromatin folding in a chromosome-dependent manner, resulting in variable levels of chromatin decompaction near telomeric regions.

### Combined analysis with telomere Hi-C showed trend of genes upregulation and TAD insulation decrease near telomeric ends

**Figure 4.**
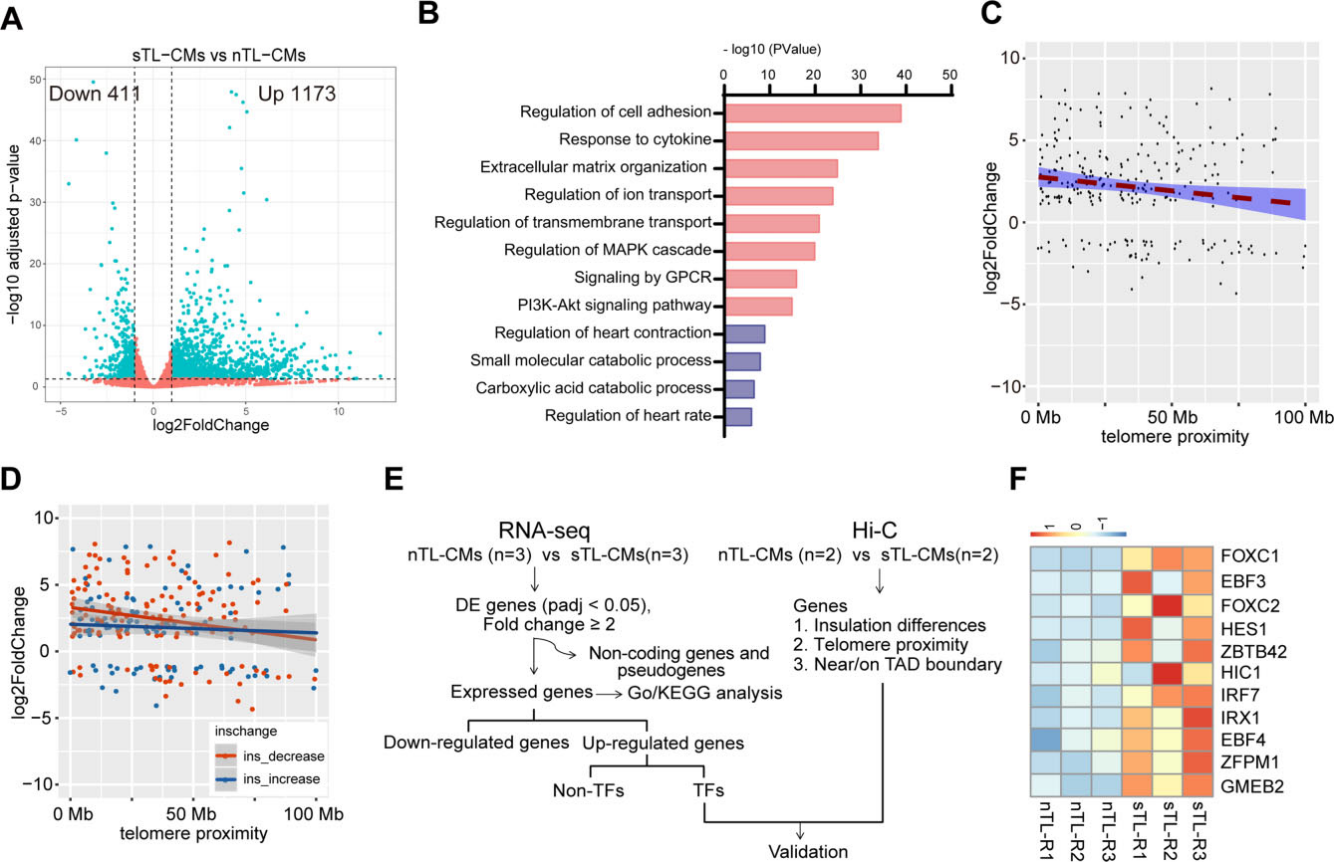
3D genomic alterations drive transcriptomic changes in sTL-CMs. (**A**) Volcano plot of differentially expressed genes between nTL- and sTL-CMs. (**B**) KEGG pathway enrichment analysis. The red bars represent pathways enriched in upregulated genes and purple represent down-regulated pathways. (**C**) Relation of changed genes (sTL-CMs versus nTL-CMs) and telomeric ends proximity. (**D**) Differentially expressed genes based on RNA-seq were mapped against TAD insulation changes relative to telomeric end proximity. (**E**) Bioinformatic processing to identify upregulated transcription factors due to genomic insulation decrease in sTL-CMs. (**F**) Top up-regulated transcriptional factors <5 Mb proximal to telomeric end and are located near TAD boundary.

To further understand the implications of chromatin decompaction in sTL-CMs, we performed RNA-seq analyses on nTL- and sTL-CMs (1173 genes upregulated and 411 genes downregulated in sTL-CMs; Figure 4A). Among DE (differentially expressed) genes, KEGG (Kyoto Encyclopedia of Genes and Genomes) pathway analysis showed significant enrichment in genes associated with response to cytokine, regulation of ion transport, oxidative phosphorylation and cell adhesion (Figure 4B and Supplementary Figure S12A–D). Interestingly, by analyzing genomic position of DE genes, we found that genes closer to telomeric ends were more upregulated (Figure 4C). Moreover, within 5 Mb proximal to telomeric ends, the average log_2_ foldchange in gene expression is 2.708812 (sTL-CMs vs nTL-CMs), with a median of 2.927773, implying an approximately 7-fold average upregulation of gene expression in the terminal 5Mb range of sTL-CMs. By mapping differentially expressed (DE) genes against TAD insulation changes relative to telomere proximity, we found that upregulated genes were more prevalent near telomeric ends with decreased TAD insulation (Figure 4D).

Among short telomere induced gene candidates that resides at telomere proximal regions, we identified and validated three genes with obvious myocardial functions: SCN7A (sodium voltage-gated channel alpha subunit 7; abnormal electrophysiology), ENO1 (enolase 1; mitochondrial dysfunction) and ATL3 (atlastin GTPase 3; aging) (Supplementary Figure S13A and B). Next, we asked whether there are transcriptional networks that is activated in sTL-CMs that is responsible for upregulation of genes at reside at non-telomeric regions. We filtered our gene candidates for transcription factors (TFs) and ranked according to expression differences (Figure 4E). Interestingly, two of our top ten TFs belong to the forkhead box protein family FOXC1 and FOXC2 (Figure 4F). We also implemented Hi-C heatmaps and included pertinent tracks regarding insulation profiles, H3K27ac and H3K27me3 surrounding the FOXC1 locus (Supplementary Figure S14). The Hi-C heatmaps indicate that FOXC1 is located near a TAD boundary. Notably, telomere shortening resulted in decreased interactions near the diagonal of Hi-C heatmaps (more blue and less red coloring in sTL-CMs) across this genomic neighborhood. Concurrently, the insulation scores at this region exhibited an overall decrease, resulting in negative insdiff values. These observed patterns are concordant with the genome-wide patterns for all TAD boundaries located in telomere proximal regions, as illustrated by the pileup heatmaps and insulation profiles in Figure 3H.

**Figure 5.**
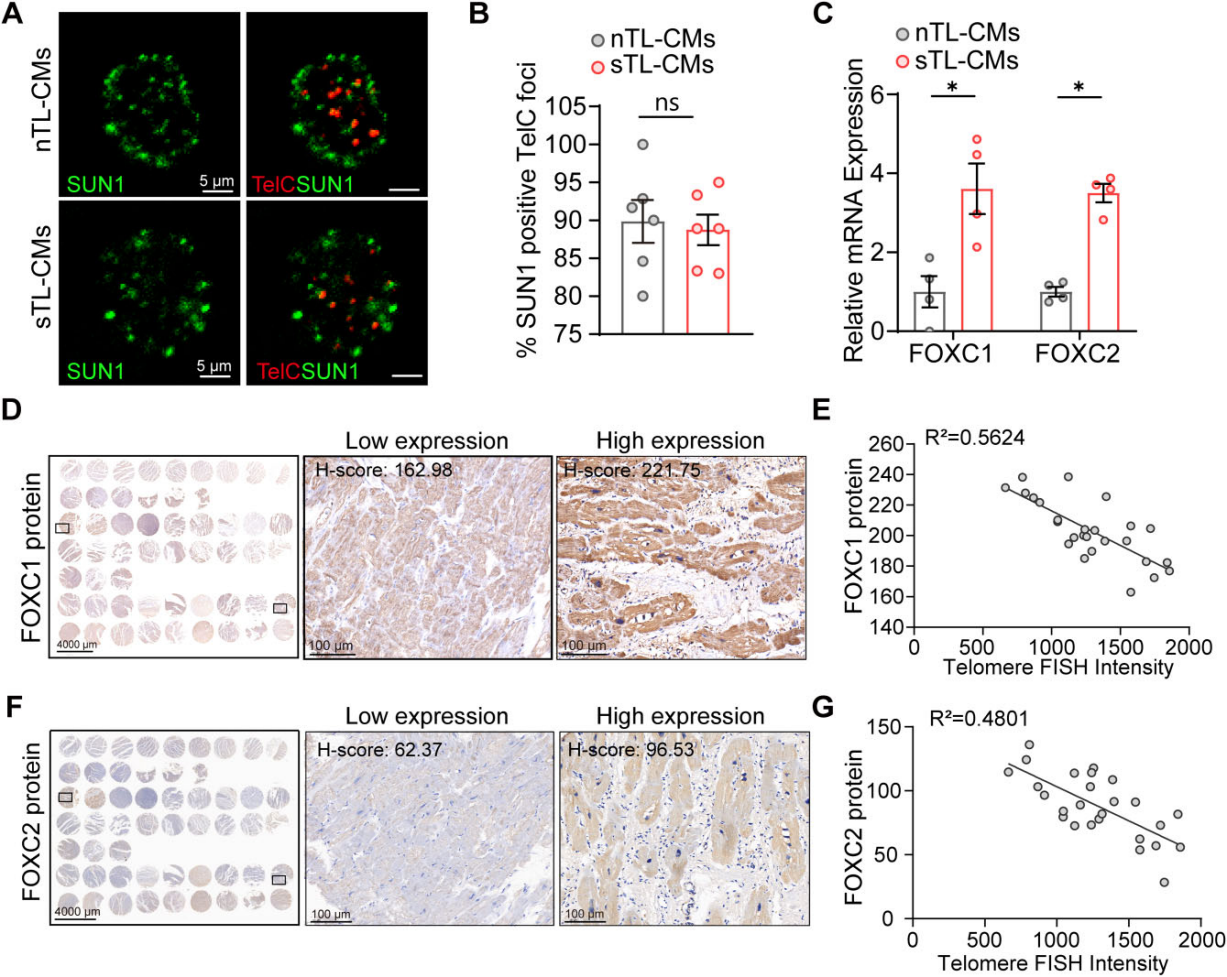
Short telomeres induce FOXC1 and FOXC2 expression in human heart biopsies. (**A, B**) Representative micrographs of hiPSC-CMs co-stained for SUN1 and telomeres showed co-localization of SUN1 at telomeric ends (biological, *n* = 6). (**C**) RT-qPCR validation of FOXC1 and FOXC2 mRNA expression. (**D**) Representative images of FOXC1 protein expression in heart failure biopsies. (**E**) Correlative analysis of FOXC1 protein levels and telomere lengths in heart biopsies. (**F**) Representative images of FOXC2 protein expression in heart biopsies. (**G**) FOXC2 protein levels negatively correlate with telomere lengths. *P≤ 0.05. Data are presented as mean values ± SEM.

Given that telomeric ends can bind to LINC complex via SUN1 (35) and we didn’t detect a difference between nTL- and sTL-CMs (Figure 5A and B), our data support a model by which short telomeres causes loss of TAD and insulation structures at telomere proximal regions and results in gene upregulation.

### FOXC1/FOXC2 expression level correlated with telomere length and aging in patient heart biopsies

It has been shown that FOXC2 share 90% sequence and 98% forkhead domain conservation to FOXC1 (50). FOXC1 and FOXC2 mRNA expression in sTL-CMs was significantly upregulated compared to nTL-CMs (Figure 5C). Further, to verify the correlation between telomere length and FOXC1/FOXC2, we performed TelC Q-FISH and immunohistochemistry staining on patient heart tissue chips consist of DCM (dilated cardiomyopathy), ICM (ischemic cardiomyopathy) and CHD (coronary atherosclerotic heart disease) biopsies (Figure 5D and F). In keep with our hiPSC-CM observations, telomere signal negatively correlated with FOXC1 and FOXC2 protein expressions in human heart failure biopsies (Figure 5E and G). We further analyzed correlation of heart biopsies telomere length from healthy and NYHA (New York Heart Association) II–IV samples. The result showed that telomeres are shorter as heart disease worsen (Supplementary Figure S15A). Moreover, FOXC1 and FOXC2 protein levels correlated positively in heart failure biopsies (Supplementary Figure S15B). To evaluate if FOXC1/FOXC2 levels coincide with cardiac aging, p21 protein levels were evaluated (Supplementary Figure S15C). In accordance, p21 levels positively correlated with FOXC1 and FOXC2 protein levels (Supplementary Figure S15D and E). These results confirm the negative correlation between telomere length and FOXC1/FOXC2 in heart failure biopsies.

### Reactivation of FOXC1 induces myocardial dysfunction and senescence

Our previous results showed the most obvious changes in FOXC1 expression in sTL-CMs, to test if FOXC1 induction in sTL-CMs is responsible for the upregulation of genes residing at telomere distal regions, we performed FOXC1 CUT&TAG sequencing (Supplementary Figure S16A and B). By pathway analysis, FOXC1 regulated genes show an enrichment in aging and degenerative diseases pathways as well as cardiac muscle contraction (Supplementary Figure S16C and D).

**Figure 6.**
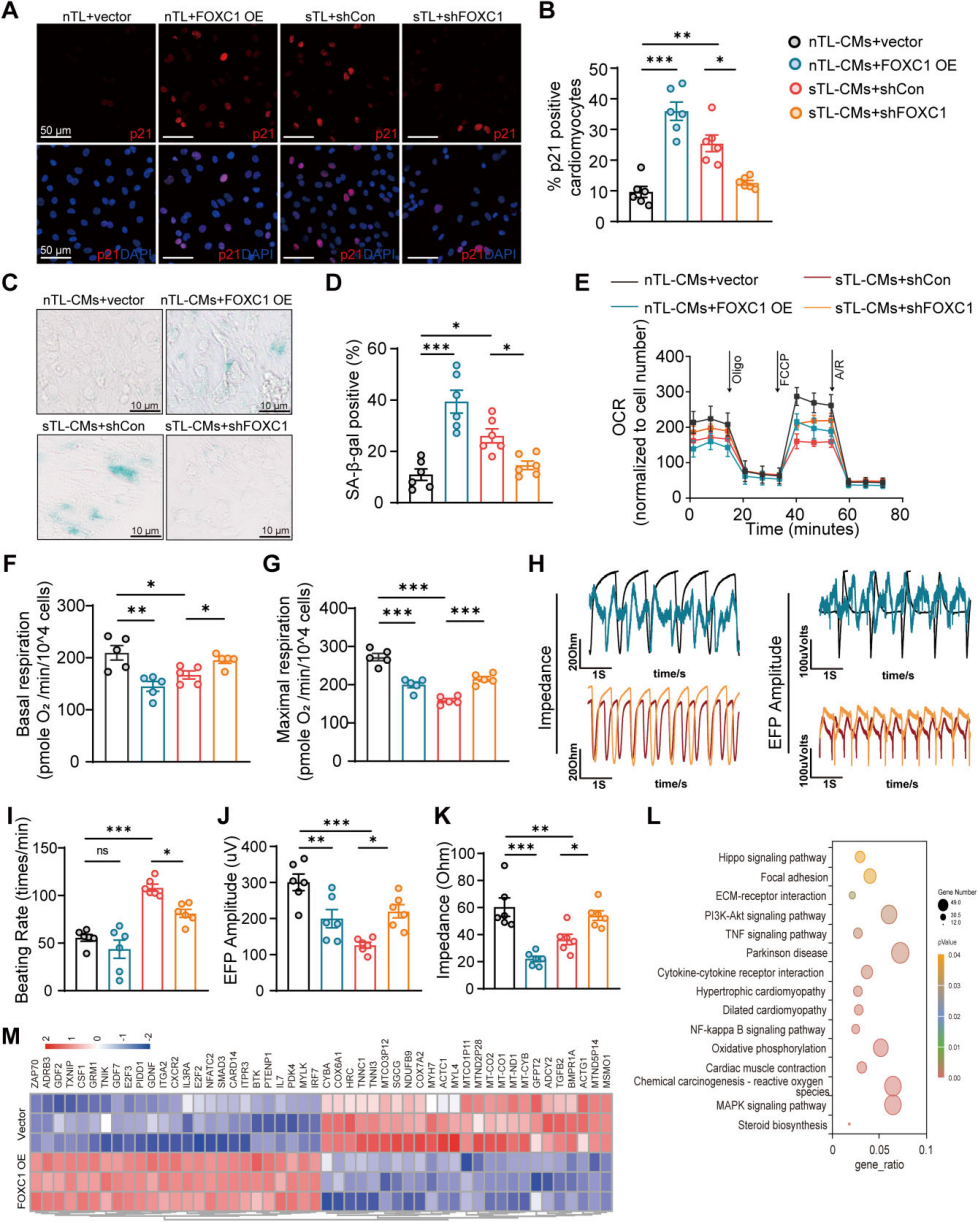
FOXC1 expression drives cardiac senescence and contractile dysfunction. (**A, B**) P21 protein expression changes in vector/FOXC1 OE nTL-CMs and shCon/shFOXC1 sTL-CMs (biological, *n* = 6). (**C, D**) SA-β-gal activity and quantification in vector or FOXC1 OE treated nTL-CMs and shCon or shFOXC1 treated sTL-CMs (biological, *n* = 6). (**E–G**) Real-time mitochondrial respiration measurements and quantification of basal mitochondrial respiration and maximal respiration of vector/ FOXC1 OE nTL-CMs and shCon/ shFOXC1 sTL-CMs (biological, *n* = 5). (**H**) Representative impedance (IMP) and extracellular field potential (EFP) traces of nTL-CMs treated with vector or FOXC1 OE and sTL-CMs treated with shCon or shFOXC1 are shown. (**I–K**) Quantification of beating rates, EFP, and IMP of vector/FOXC1 OE nTL-CMs and shCon/shFOXC1 sTL-CMs (biological, *n* = 6). (**L**) Kyoto Encyclopedia of Genes and Genomes enrichment analysis show top 15 changed pathways in FOXC1 OE nTL-CMs compared to vectors. (**M**) Heatmap of genes that are differently expressed in FOXC1 OE nTL-CMs; genes are by column and samples by row. *P≤ 0.05, **P≤ 0.01 and ***P≤ 0.001. Data are presented as mean values ± SEM.

Next, we asked if FOXC1 induction in sTL-CMs is responsible for declined contractile and mitochondrial function. First, FOXC1 mRNA expression levels declined as cardiac differentiation proceeded in both nTL- and sTL-CMs (Supplementary Figure S17A). Next, we overexpressed FOXC1 (FOXC1 OE) in nTL-CMs as well as shRNA knocked down FOXC1 (shFOXC1) in sTL-CMs using lentivirus (Supplementary Figure S17B). Overexpression of FOXC1 in nTL-CMs, resulted in a significant increase in percentage of p21/p16 (Figure 6A and B, Supplementary Figure S17C and D) and SA-β-gal positive CMs (Figure 6C and D). Reversibly, shFOXC1 knockdown in sTL hiPSC-CMs ameliorated myocardial senescence (Figure 6A-D, Supplementary Figure S17C and D). Expression of aging-related genes CDKN2A and TP53 were induced in FOXC1-OE nTL-CMs and reversed in shFOXC1 sTL-CMs (Supplementary Figure S17E). Furthermore, FOXC1 overexpression in nTL-CMs resulted in reduced mitochondrial respiration; whereas, shFOXC1 improved mitochondrial respiratory capacity in sTL-CMs (Figure 6E–G). In accordance, FOXC1 overexpression in nTL-CMs resulted in contractile dysfunction marked by decrease in contractility and EFP; shFOXC1 restored contractile function in sTL-CMs (Figure 6H–K). To further characterize effect of FOXC1 overexpression on myocardial transcriptome, RNA-seq was performed on nTL-CMs overexpressing FOXC1. Compared to control, FOXC1 overexpression resulted in an up-regulation of inflammation-associated genes and a significant down-regulation of cardiac muscle contraction and mitochondrial oxidative respiration related genes (Figure 6L, M). Together, these results demonstrate that upregulation of FOXC1 drives myocardial senescence, mitochondrial dysfunction, and contractile dysfunction.

## Discussion

This work revealed telomere shortening triggers chromosomal decompaction near telomeric ends and through upregulation of FOXC1 drives senescence in sTL-CMs. Using Hi-C we demonstrate that short telomeres cause chromatin decompaction near telomeric ends but does not perturb telomere-SUN1 co-localizations suggesting telomeric stretching in sTL-CMs. These observations were further confirmed by increase in H3K27me3 and H3K27ac marks and upregulation of transcription near telomeric ends. Through bioinformatic analysis, we identified upregulation of FOXC1 in sTL-CMs and through overexpression in nTL-CMs and shFOXC1 knockdown in sTL-CMs we demonstrate that induction of FOXC1 drive myocardial aging.

Telomeric ends (51) and interstitial telomeric sequences can bind to lamin A/C via TRF2 (52,53) and is thought to anchor the chromosomal ends with some mobility (54).We suspect that this mobility and/or telomere anchorage help define the chromatin landscape through telomere-lamin A/C tether and disruption can drive accelerated aging in cardiomyocytes. In support, pathogenic mutations in lamin A/C (55) (LMNA/C) cause abnormal nuclear structure, disrupt peripheral chromatin (53), abnormal nuclear pore opening which results in elevated DNA damage responses (56,57) and lamin A/C uncoupling through overexpression of dominant-negative SUN1 prevents laminopathic heart failure in mice (58). Heterochromatins have been shown to locate and bind at nuclear lamina for silencing that is sensitive to mechanical perturbation (59). Here we demonstrate that in sTL-CMs, chromosomal decompaction near telomeric ends perhaps serve as a different kind of mechanical perturbation to chromatin structure. However, given that we do not know the degree of telomere shortening per chromosomal arms, we were unable to determine if telomeric ends shorten differently and whether such process affect chromatin structure is cell-type specific. As telomere-to-telomere results are now available (60,61), it would be interesting to examine if telomere shortening rate variation and subsequent chromatin decompaction is cell-type and chromosomal specific.

FOXC1 belongs to the Forkhead box (FOX) family of transcription factors marked by evolutionary conserved fork-head or ‘winged-helix’ DNA-binding domain. FOXC1 plays a critical role in cardiogenesis (62–64) and cardiac pathologies (65,66). Molecularly, we show that contrary to DNA damage-induced cardiac dysfunction (67), overexpression of FOXC1 in nTL-CMs sufficiently induced myocardial senescence, induced inflammation-associated genes while downregulated sarcomeric and mitochondrial genes. Conversely, shFOXC1 knockdown in sTL-CMs was sufficient in blocking senescence and restoring mitochondrial function. Importantly, we confirmed that FOXC1 protein levels were induced and negatively correlated with myocardial telomere length in heart failure patient hearts. Molecularly, we demonstrate that FOXC1 protein upregulation drives the transcription of 284 out of 1584 differentially expressed genes in sTL-CMs compared to nTL-CMs.

In keep with our previous findings (20,28), sTL-CMs were associated with DNA damage response and cellular senescence. Not only did we observe an increase in p21^CIP^ and p16^Ink4a^ levels in sTL-CMs, but sTL-CMs also exhibited abnormal electrophysiology and mitochondrial dysfunction. Aside from its complex homeostatic regulations (68) and emerging evidence of intercellular mitochondria transfer (69,70), mitochondria have been shown to be necessary for cells to enter senescence (71). Although short telomeres have been shown to induce mitochondrial dysfunction through the p53-PGC1a signaling pathway (26,28), we demonstrate that overexpression of FOXC1 protein in nTL-CMs alone was sufficient in inducing myocardial senescence and mitochondrial dysfunction while shFOXC1 in sTL-CMs ameliorates.

Aged hearts exhibit structural, electrical and functional changes including increased ventricular wall thickening, myocardial fibrosis, diastolic dysfunction and progressive decline in cardiac reserve (72,73), atrial fibrillation, and cardiac ischemia (74,75). Leucocyte telomere lengths (LTLs) are negatively correlated with cardiovascular disease risks (76–78). While tissue telomere lengths are highly correlated with LTLs (79), we speculate that systemic aging (LTL shortening) is more likely to affect vascular endothelium while myocardial telomere shortening drives aging from within. As we demonstrate the role of telomere length in post-mitotic myocardial aging, we speculate the same mechanisms may be present in other post-mitotic tissues (12).

In summary, the chromatin remodeling induced by short telomeres revealed in this work paves the way for cellular aging studies involving telomere shortening, DNA damage response, and transcription network regulation. Such information should shed light on the biomechanics-function relationship of telomeres in the context of aging in post-mitotic tissues.

## Supplementary Material

gkae274_Supplemental_File

## Data Availability

All data have been included in the manuscript, figures and supplemental data. Data supporting finding of this study are available from corresponding authors upon reasonable request. All sequencing data reported in this study have been uploaded to GEO database with an accession number GSE217703.
