# Supplementary material for: Proximal telomeric decompaction due to telomere shortening drives FOXC1-dependent myocardial senescence
Source: Nucleic Acids Res. 2024 Apr 18;52(11):6269–84. doi: 10.1093/nar/gkae274 (PMC11194093; doi:10.1093/nar/gkae274)
Supplement: gkae274_Supplemental_File [file gkae274_supplemental_file.pdf]

Supplementary Data

Proximal telomeric decompaction due to telomere shortening drives FOXC1-dependent myocardial senescence

Supplementary Figure S1-S17
Supplementary Table S1-S3

FIGURES AND FIGURE LEGENDS

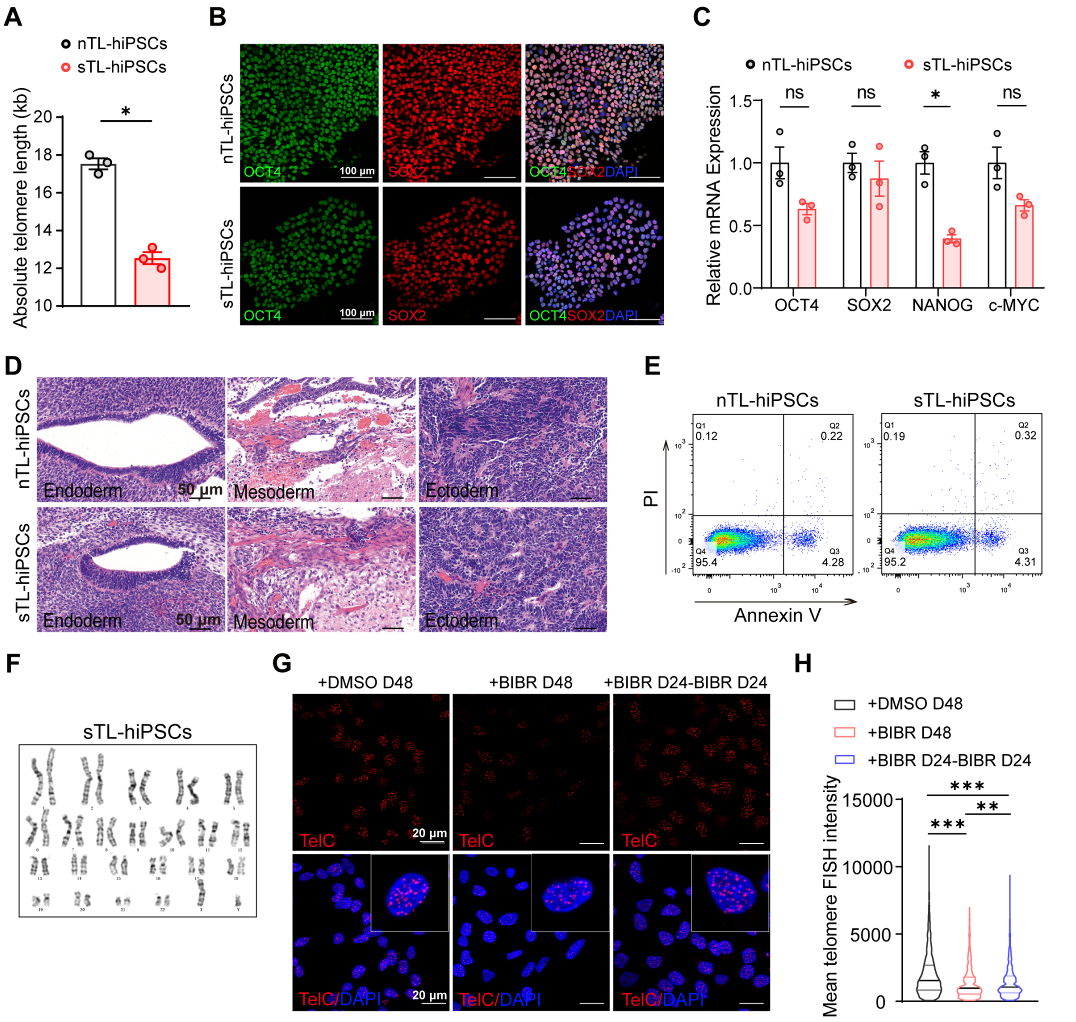

Figure S1: Generation of isogenic short telomere hiPSCs. (A) Absolute mean telomere length of nTL- and sTL-hiPSCs measured by RT-qPCR (biological, n = 3). **(B)** Representative images of immunofluorescence staining of the pluripotent markers OCT4 and SOX2 in nTL- and sTL-hiPSCs. **(C)** Relative mRNA expression levels of pluripotency, genes NANOG, OCT4, SOX2 and c-MYC in nTL- and sTL-hiPSCs (biological, n = 3). **(D)** Pluripotency of nTL- and sTL-hiPSCs were evaluated using the teratoma assay and of endodermal, mesodermal, and ectodermal layers are shown. **(E)** Flow cytometry analysis of apoptosis in nTL- and sTL-hiPSCs. **(F)** Karyotype of sTL-hiPSCs. **(G-H)** Reversibility of telomere shortening due to BIBR treatment evaluated by Q-FISH in hiPSCs (n = 80 cells per group, biological, n = 2). D48 and D24 indicate day 24 and day 48 from the first day of BIBR1532 treatment (Day1). +BIBR: hiPSCs cultured with BIBR1532; -BIBR: hiPSCs cultured without BIBR1532. *$P \leq 0.05$, **$P \leq 0.01$ and ***$P \leq 0.001$. Data are presented as mean values ± SEM.

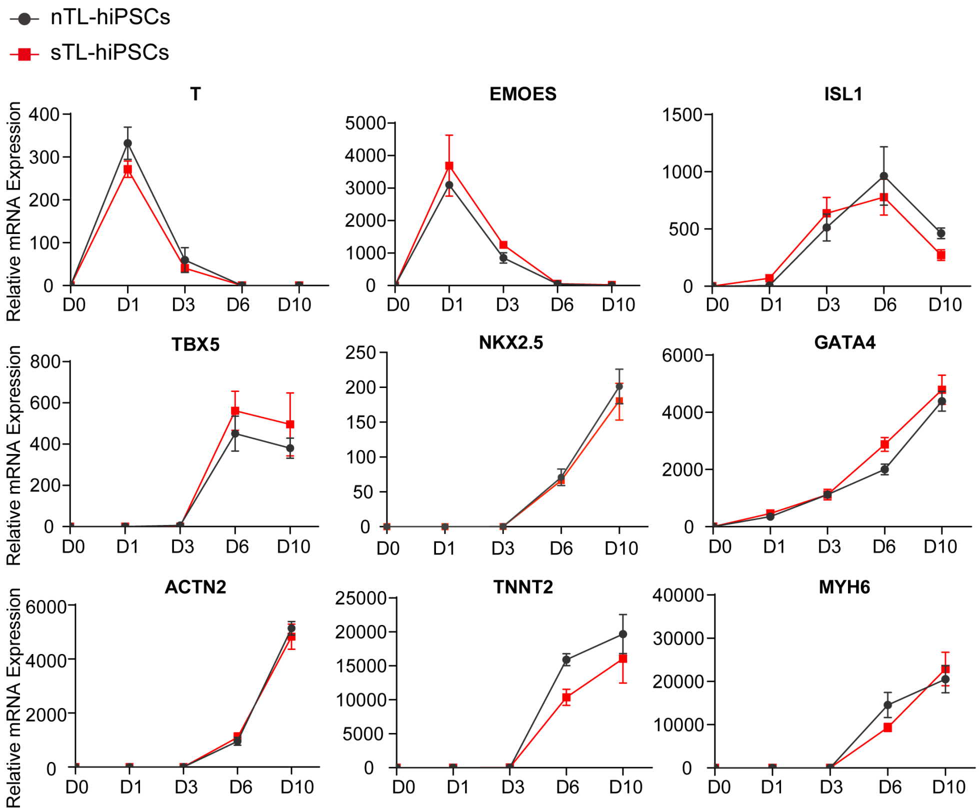

Figure S2: Cardiac differentiation efficiency of nTL- and sTL-hiPSCs. Expression of cardiac lineage commitment genes during hiPSC-CM differention evaluated at Day0, Day1, Day3, Day6, and Day10 by RT-qPCR (biological, n = 4). Data are presented as mean values ± SEM.

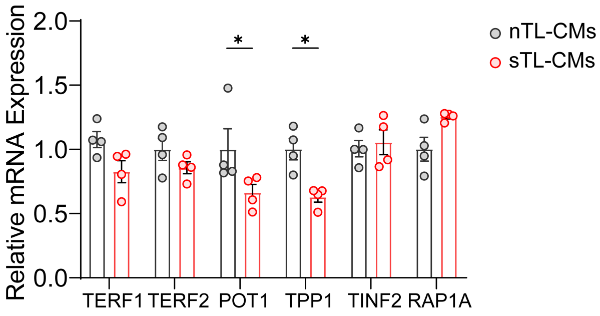

Figure S3: Shelterin expression in nTL- and sTL-CMs. Expression of shelterin genes in nTL- and sTL-CMs were evaluated by RT-qPCR (biological, n = 4). *$P \leq 0.05$. Data are presented as mean values ± SEM.

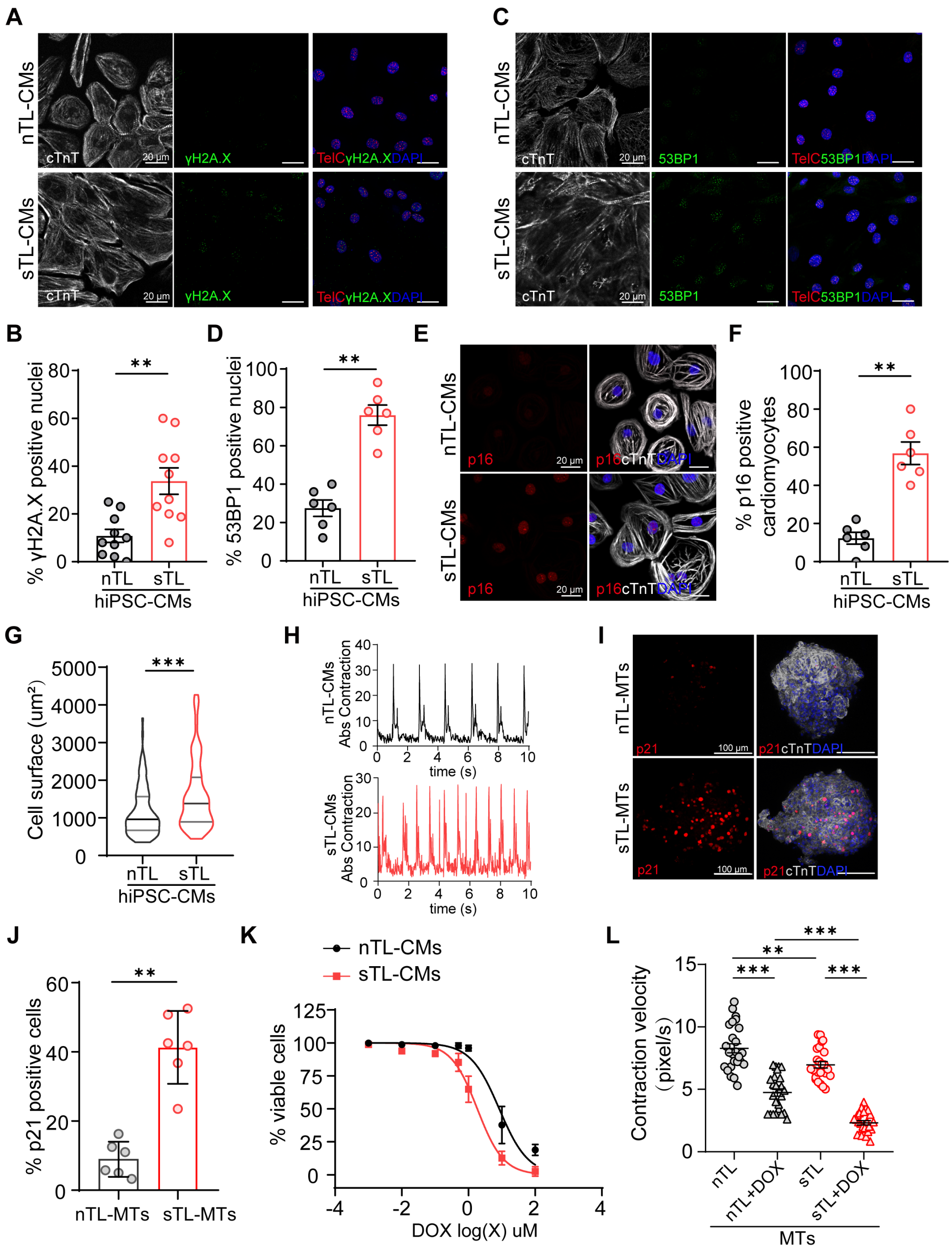

Figure S4: STL-CMs exhibit increased DNA damage response and myocardial senescence. (A-B) Representative micrographs and quantification of γH_2A.X positive hiPSC-CM nuclei (nTL-CMs: n = 103 cells; sTL-CMs: n = 98 cells, biological, n = 3, every dot represents the averaged value from technical replicates). **(C-D)** Representative micrographs and quantification of 53BP1 positive hiPSC-CM nuclei (nTL-CMs: n = 65 cells; sTL-CMs: n = 73 cells, biological, n = 3, every dot represents the averaged value from technical replicates). **(E-F)** Representative micrographs and quantification of p16-positive hiPSC-CMs (nTL-CMs: n = 50 cells; sTL-CMs: n = 45 cells, biological, n = 3, every dot represents the averaged value from technical

replicates). **(G)** Quantification of cell surface area (um^2) (nTL-CMs: n = 116 cells; sTL-CMs: n = 127 cells, biological, n = 3). **(H)** Representative motion traces of single nTL- and sTL-CMs are shown. **(I-J)** Representative micrographs and quantification of p21 levels in nTL- and sTL-MTs. biological, n = 3, every dot represents the averaged value from technical replicates. **(K)** Vulnerability of doxorubicin (DOX) cytotoxicity for nTL-CMs and sTL-CMs were evaluated 48 h post doxorubicin challenge in a dose titration manner. IC50 were extrapolated for nTL-CMs (8.36 μM) and sTL-CMs (1.84 μM) from three independent experiments. A minimum of 10 fields of view were analysed for each concentration. **(L)** Contraction velocity of nTL- and sTL-MTs before and after 0.5 μM doxorubicin challenge are shown (n > 15 MTs per group, biological, n = 2). *$P \leq 0.05$, **$P \leq 0.01$ and ***$P \leq 0.001$. Data are presented as mean values ± SEM.

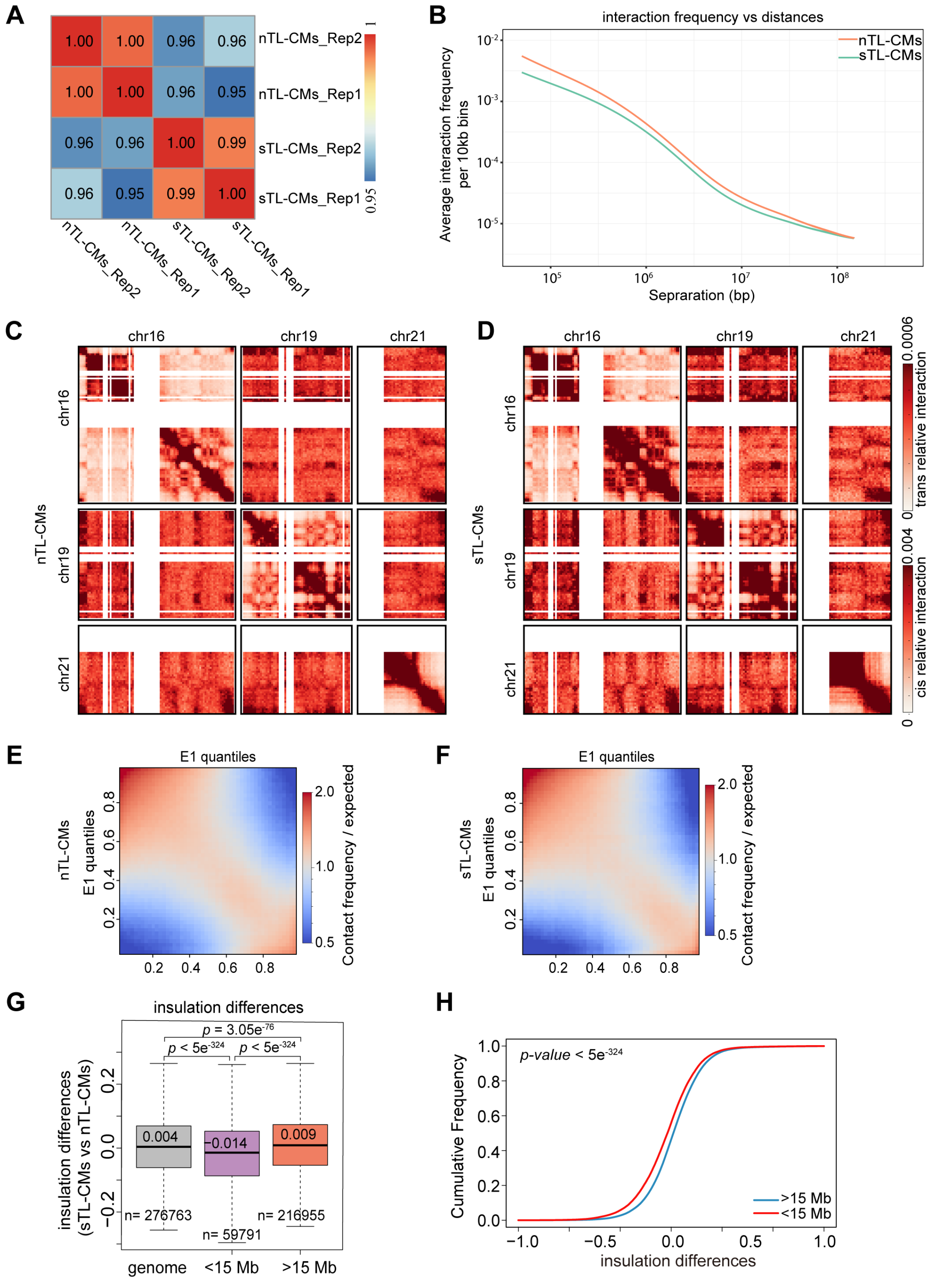

Figure S5: Chromatin conformation changes due to shortened telomeres in hiPSC-CMs. (A) Correlation heatmap of pairwise combinations of Hi-C datasets. Biological replicates for each condition are highly correlated. **(B)** *P(s)* curves indicate relationships between chromatin contact probability and genomic distances for chromatin interactions on autosomes in nTL- and sTL-CMs. Dotted lines corresponding to P(s) ~ s−0.6 are shown as references. **(C-D)** Representative *cis* and *trans* Hi-C heatmaps of chr16, chr19 and chr21. **(E-F)** Saddle plots quantify the overall level of genome

compartmentalization of nTL- and sTL-CMs. **(G)** Insulation differences of whole genome (all) and chromatin within/over 15 Mb from telomeric ends between sTL- and nTL-CMs. grey box: whole genome; purple box: within 15 Mb from telomeric ends; orange box: over 15 Mb from telomeric ends. **(H)** Cumulative frequency plots of insulation difference in telomere-proximal (within 15 Mb) versus telomere-distal (over 15 Mb) regions.

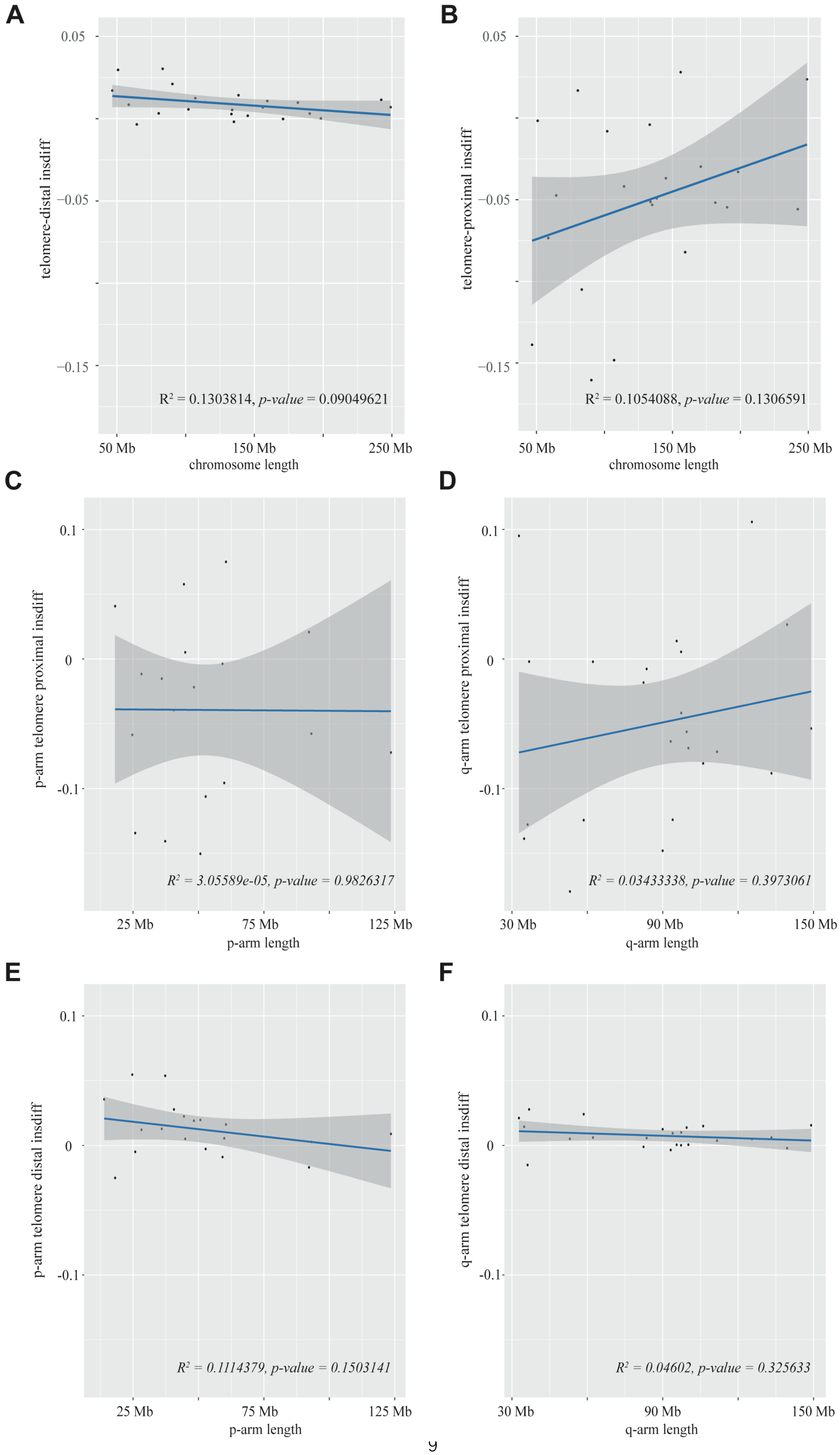

A
0.05
−0.05
−0.15
telomere-distal insdiff
R² = 0.1303814, p-value = 0.09049621
50 Mb
150 Mb
250 Mb
chromosome length
B
0.05
−0.05
−0.15
telomere-proximal insdiff
R² = 0.1054088, p-value = 0.1306591
50 Mb
150 Mb
250 Mb
chromosome length
C
0.1
0
-0.1
p-arm telomere proximal insdiff
R² = 3.05589e-05, p-value = 0.9826317
25 Mb
75 Mb
125 Mb
p-arm length
D
0.1
0
-0.1
q-arm telomere proximal insdiff
R² = 0.03433338, p-value = 0.3973061
30 Mb
90 Mb
150 Mb
q-arm length
E
0.1
0
-0.1
p-arm telomere distal insdiff
R² = 0.1114379, p-value = 0.1503141
25 Mb
75 Mb
125 Mb
p-arm length
F
0.1
0
-0.1
q-arm telomere distal insdiff
R² = 0.04602, p-value = 0.325633
30 Mb
90 Mb
150 Mb
q-arm length

Figure S6: Correlation between chromosome length and insulation differences.
(A-B) Scatter diagrams of insulation score differences (insdiff) against chromosome length plotted for telomere distal and telomere proximal regions. **(C-D)** Scatter diagram of chromosome p-arm and q-arm lengths and insulation score differences in telomere proximal regions. **(E-F)** Scatter diagram of chromosome p-arm and q-arm lengths and insulation score differences in telomere distal regions.

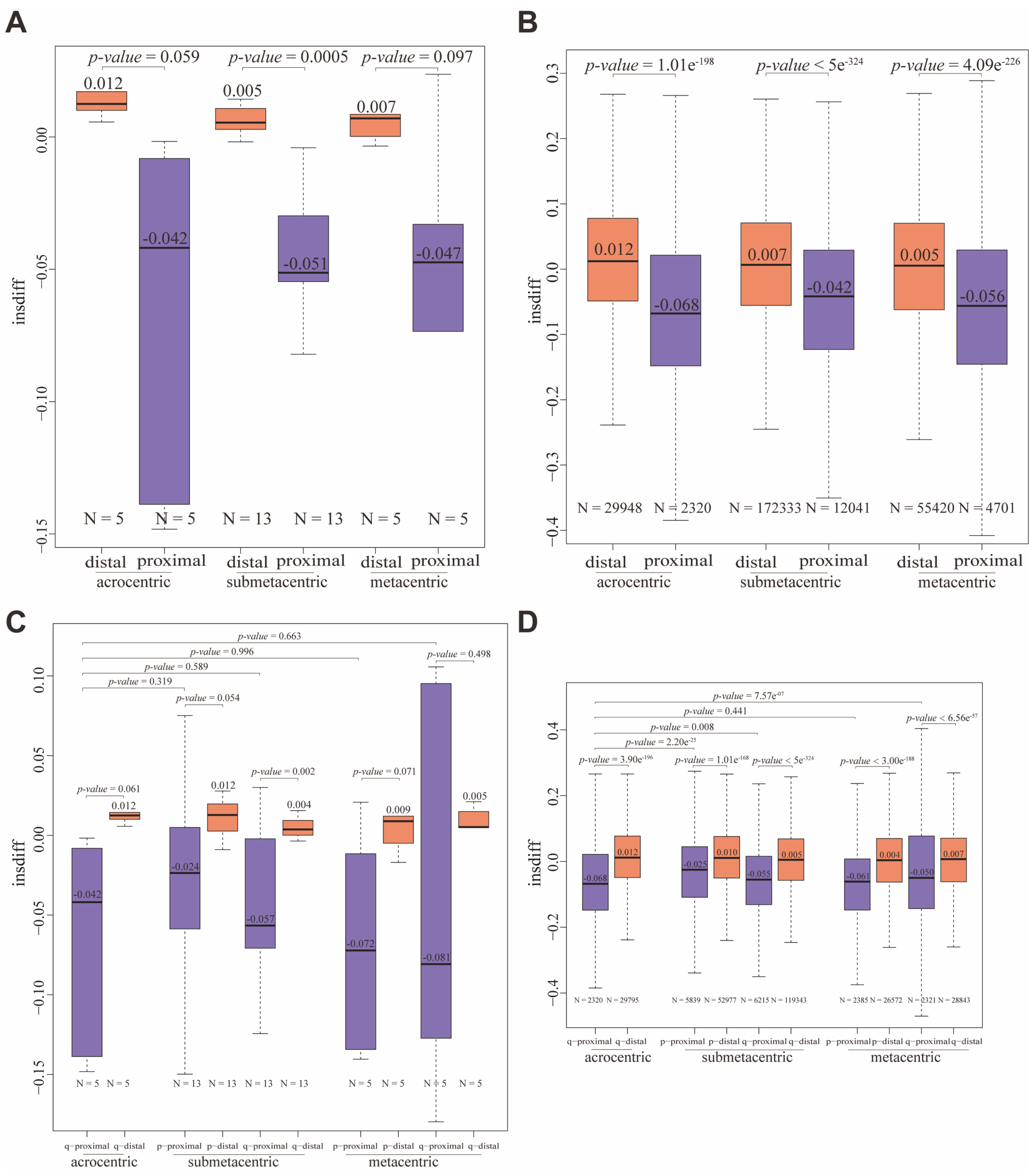

Figure S7: Acrocentric, submetacentric and metacentric chromosomes exhibited consistent trend on insulation differences between telomere-proximal and -distal regions. (A-B) Boxplot of insulation score differences (insdiff) between telomere-distal and telomere-proximal regions for acrocentric, submetacentric and metacentric chromosomes (**A**, each chromosome as a single data point; **B**, all 10 kb bins). **(C-D)** Boxplot of insulation differences between telomere-distal and telomere-proximal regions for p-arms and q-arms of acrocentric, submetacentric and metacentric chromosomes (**C**, each chromosome arm as a single data point; **D**, all 10 kb bins). The upper whisker extends from the hinge to the largest value no further than 1.5 × IQR (inter-quartile range) from the hinge, and the lower whisker from the hinge to the lowest value within 1.5 × IQR of the hinge. The values beyond the whiskers are not shown in the boxplots. N, the total number of bins identified in each condition. The N values are indicated at boxplots.

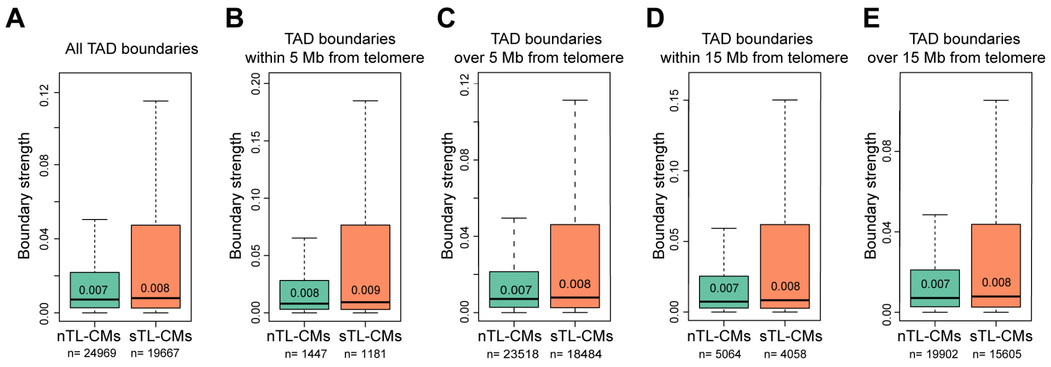

Figure S8: sTL-CMs exhibit stronger boundary strength near telomeric ends. (A-E) Box plots quantifying the strength of TAD boundaries within/over 5 Mb from telomeric ends between nTL- and sTL-CMs. The upper and lower bounds of boxes represent the third and the first quartiles of TAD boundary strength, respectively. Centre bars represent the median of TAD boundary strength. The upper whisker extends from the hinge to the largest value no further than 1.5 × IQR (inter-quartile range) from the hinge, and the lower whisker from the hinge to the lowest value within 1.5 × IQR of the hinge. The values beyond the whiskers are not shown in the boxplots. n, the total number of TAD boundaries identified in each condition. The n values are indicated at the bottom of the boxplots.

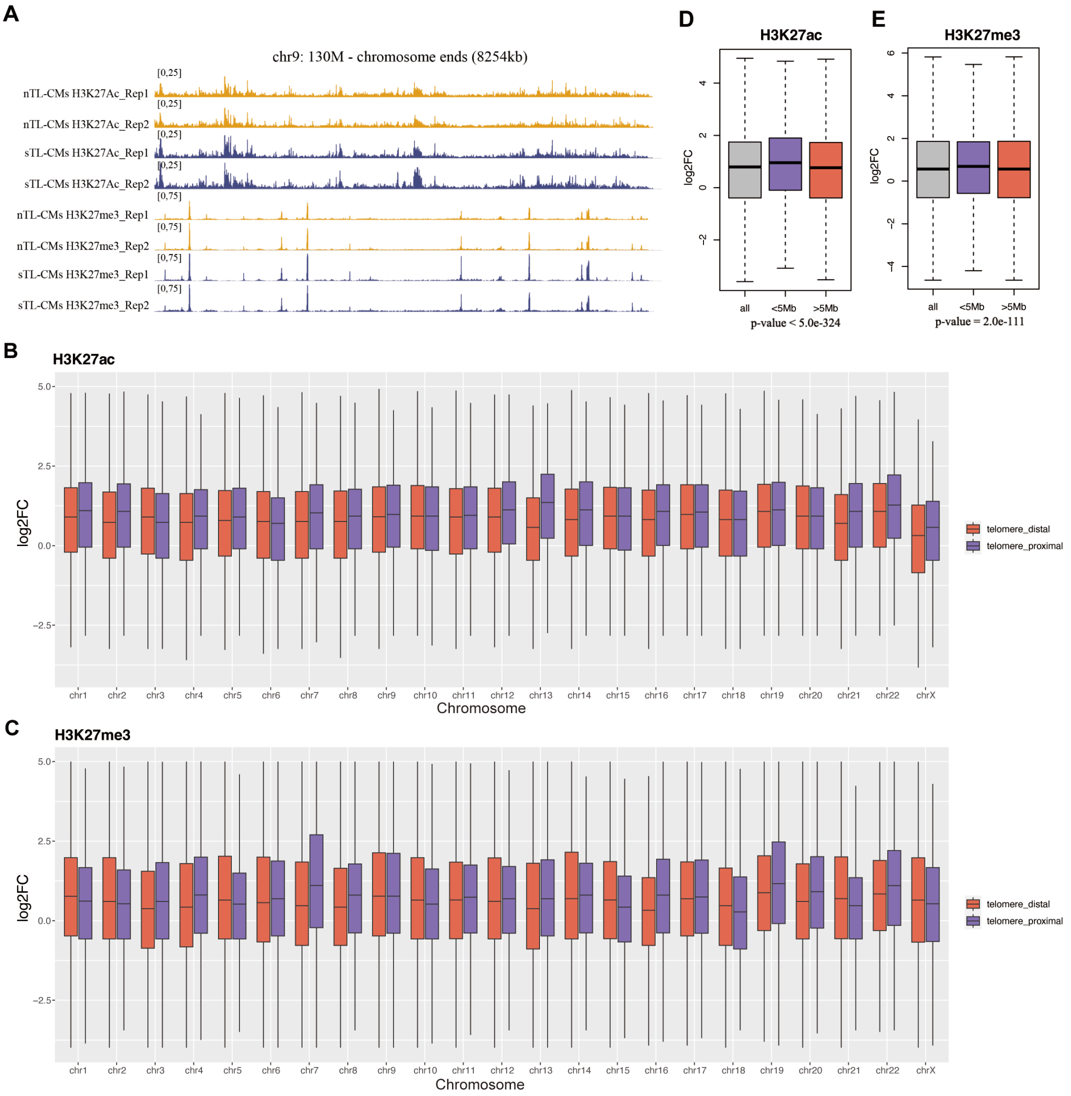

Figure S9: Enrichment of H3K27ac and H3K27me3 marks at telomere-proximal regions in sTL-CMs. (A) Representative track view showing two biological replicates of H3K27ac and H3K27me3 signal in nTL-CMs and sTL-CMs. **(B-C)** Boxplots of H3K27ac and H3K27me3 fold changes (between nTL- and sTL-CMs) on different chromosomes within/over 5 Mb from telomeric ends are shown. **(D-E)** Average H3K27ac and H3K27me3 fold enrichment are shown. Box limits, upper and lower quartiles. "all" indicates the whole genome, " < 5 Mb" indicates signal intensity within 5 Mb from telomeric ends while " > 5 Mb" indicates signal intensity over 5 Mb from telomeric ends. Centre bars, median. Whiskers, 1.5× interquartile range. log2FC: log2 fold change.

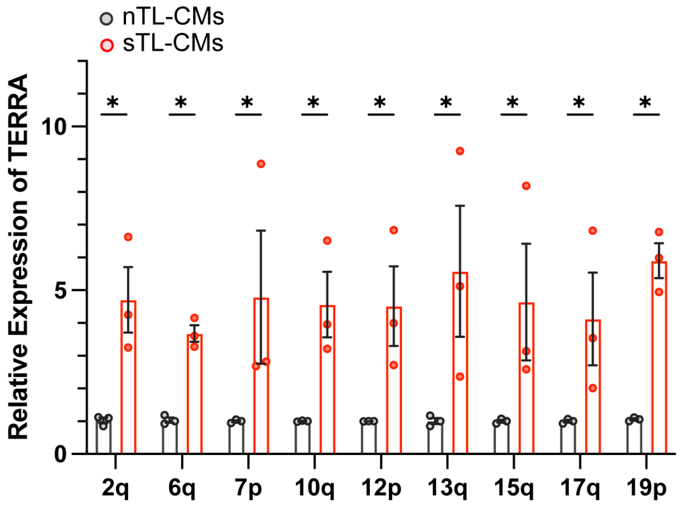

Figure S10: TERRA expression in nTL- and sTL-CMs. Relative expression of chromosomal specific TERRAs were measured using RT-qPCR. GAPDH was used for normalization. n = 3, biological. *$P \leq 0.05$. Data are presented as mean values ± SEM.

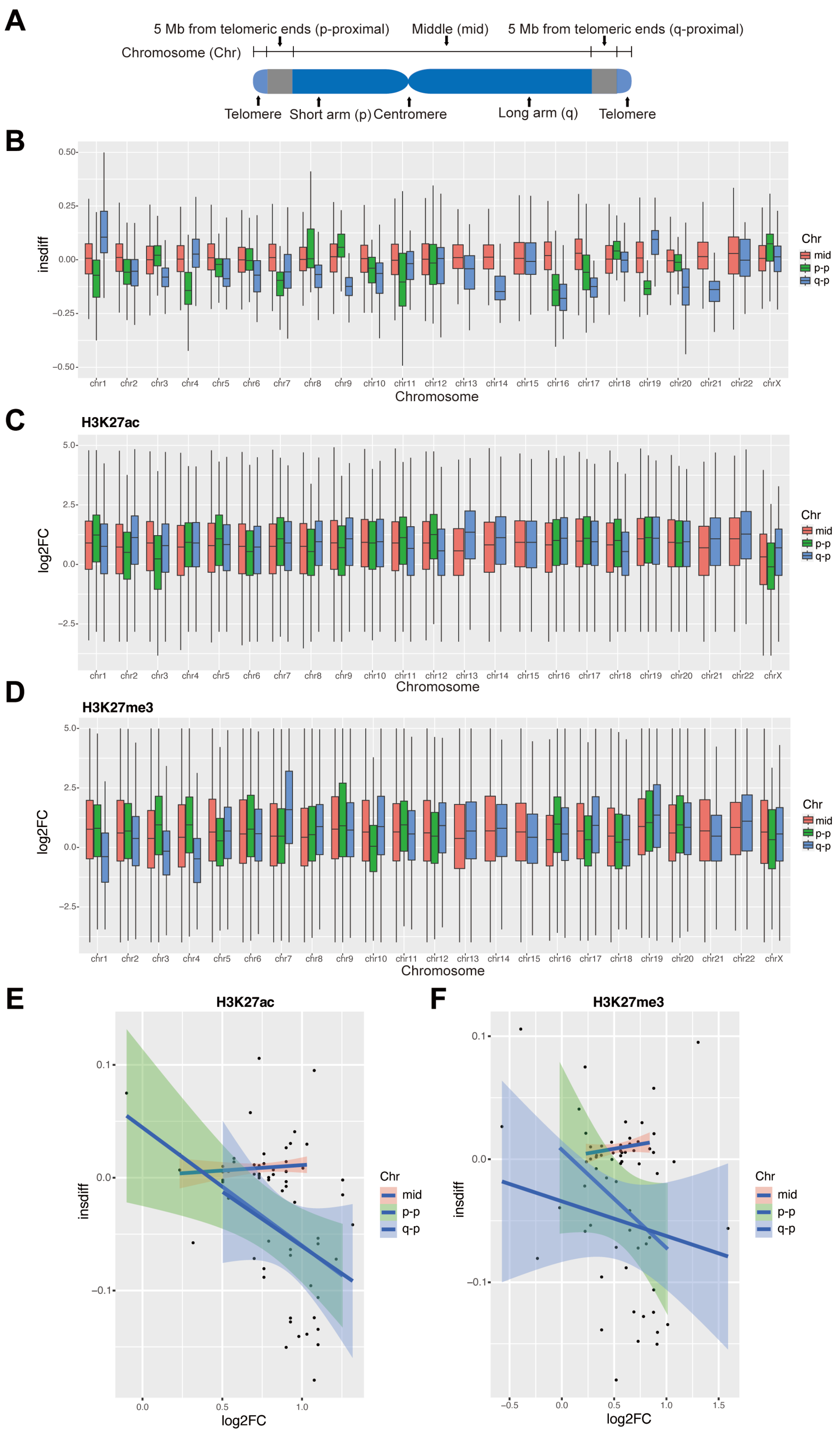
A
5 Mb from telomeric ends (p-proximal)
Middle (mid)
5 Mb from telomeric ends (q-proximal)
Chromosome (Chr)
Telomere
Short arm (p)
Centromere
Long arm (q)
Telomere
B
insdiff
Chromosome
Chr
mid
p-p
q-p
C
H3K27ac
log2FC
Chromosome
Chr
mid
p-p
q-p
D
H3K27me3
log2FC
Chromosome
Chr
mid
p-p
q-p
E
H3K27ac
insdiff
log2FC
Chr
mid
p-p
q-p
F
H3K27me3
insdiff
log2FC
Chr
mid
p-p
q-p

Figure S11: Correlation of H3K27ac/H3K27me3 fold changes and the insulation scores differences. (A) Schematic showing chromosomal binning definitions. **(B-D)** Boxplots displaying insulation scores differences, H3K27ac fold changes and H3K27me3 fold changes between nTL- and sTL-CMs calculated for individual chromosomal arms (p- and q-arms) binned for < 5 Mb (p-p and q-p) and ≥ 5 Mb (mid). mid: middle; p-p: p-proximal; q-p: q-proximal; insdiff: insulation score differences; log2FC: log2 fold change. **(E-F)** Scatter diagram of H3K27ac/H3K27me3 fold changes plotted against insulation differences for p-proximal (p-p), q-proximal (q-p) and middle (mid) regions.

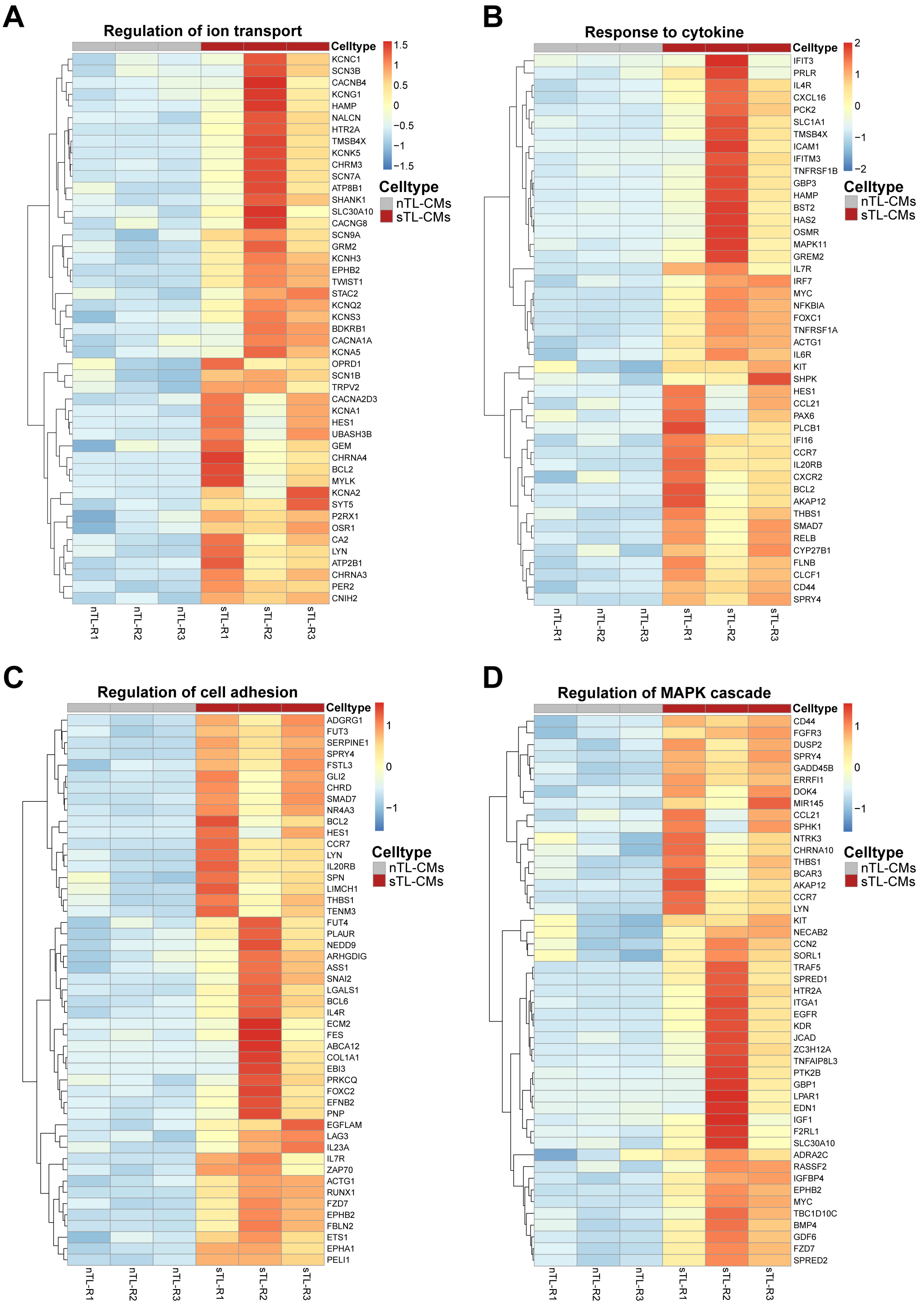

Figure S12: Pathway analyses of differentially expressed genes between nTL- and sTL-CMs. Heatmaps showing KEGG pathways of differentially expressed genes between nTL- and sTL-CMs enriched in **(A)** ion transport, **(B)** response to cytokine, **(C)** regulation of cell adhesion, and **(D)** regulation of MAPK cascade.

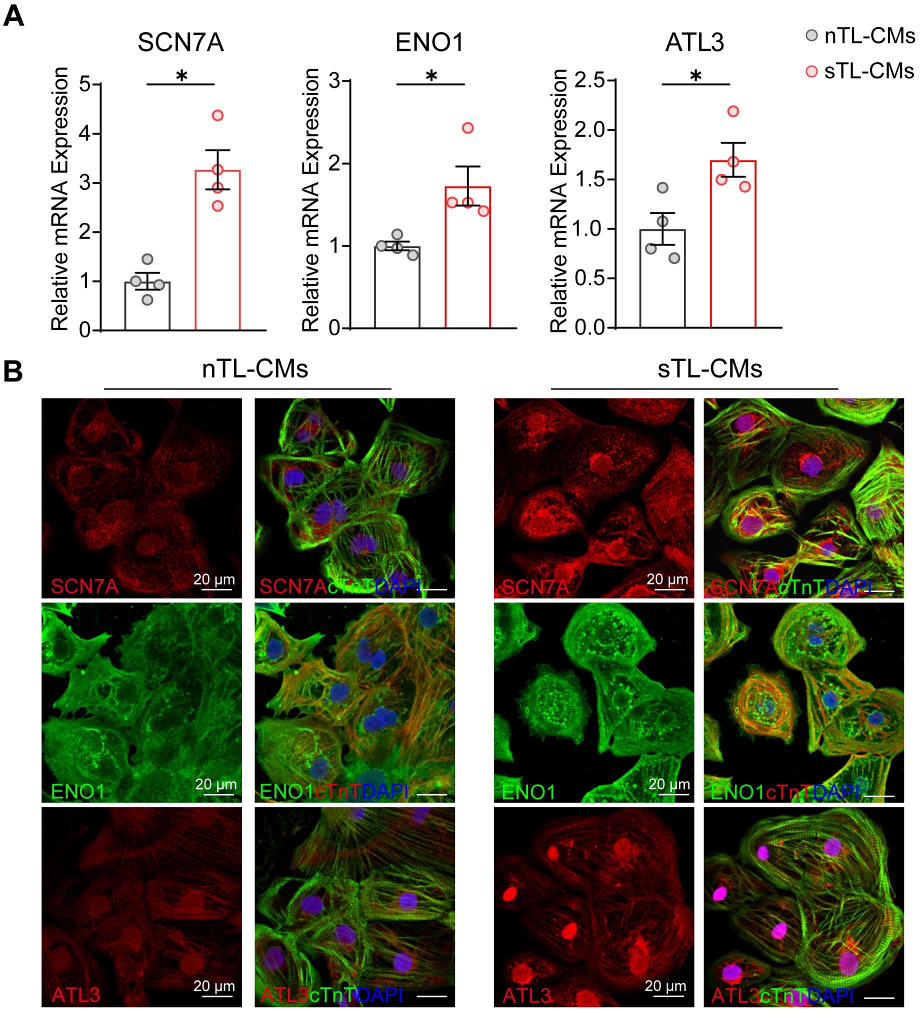

Figure S13: Identification and validation of short telomere induced genes change. **(A)** Expression of SCN7A, ENO1 and ATL3 in nTL- and sTL-CMs (biological, n = 4). **(B)** Protein expression of SCN7A, ENO1 and ATL3 in nTL- and sTL-CMs were evaluated by immunofluorescence staining. $P \leq 0.05$. Data are presented as mean values ± SEM.

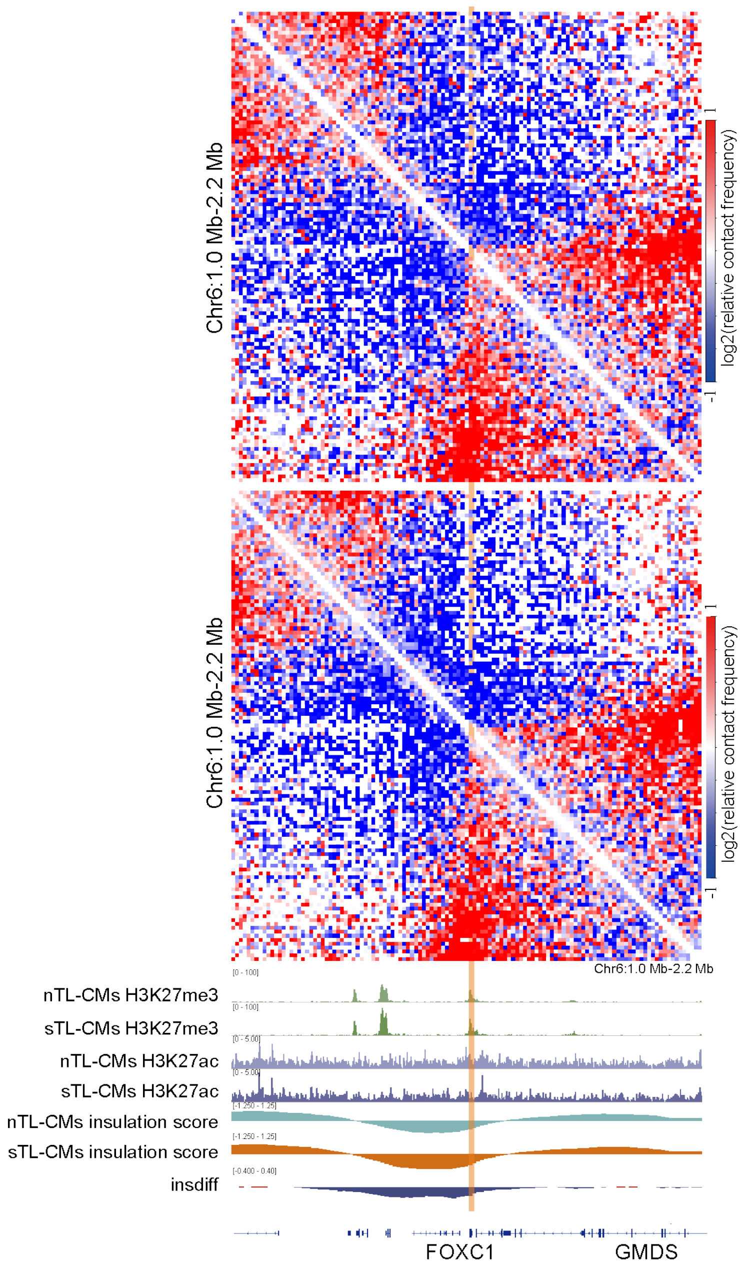

Figure S14: Hi-C heatmaps and insulation profiles, H3K27ac, and H3K27me3 tracks around the FOXC1 locus. Hi-C heatmaps and pertinent tracks regarding insulation profiles, H3K27ac and H3K27me3 surrounding the FOXC1 locus (chr6: 1,000,000-2,200,000). insdiff: insulation score differences.

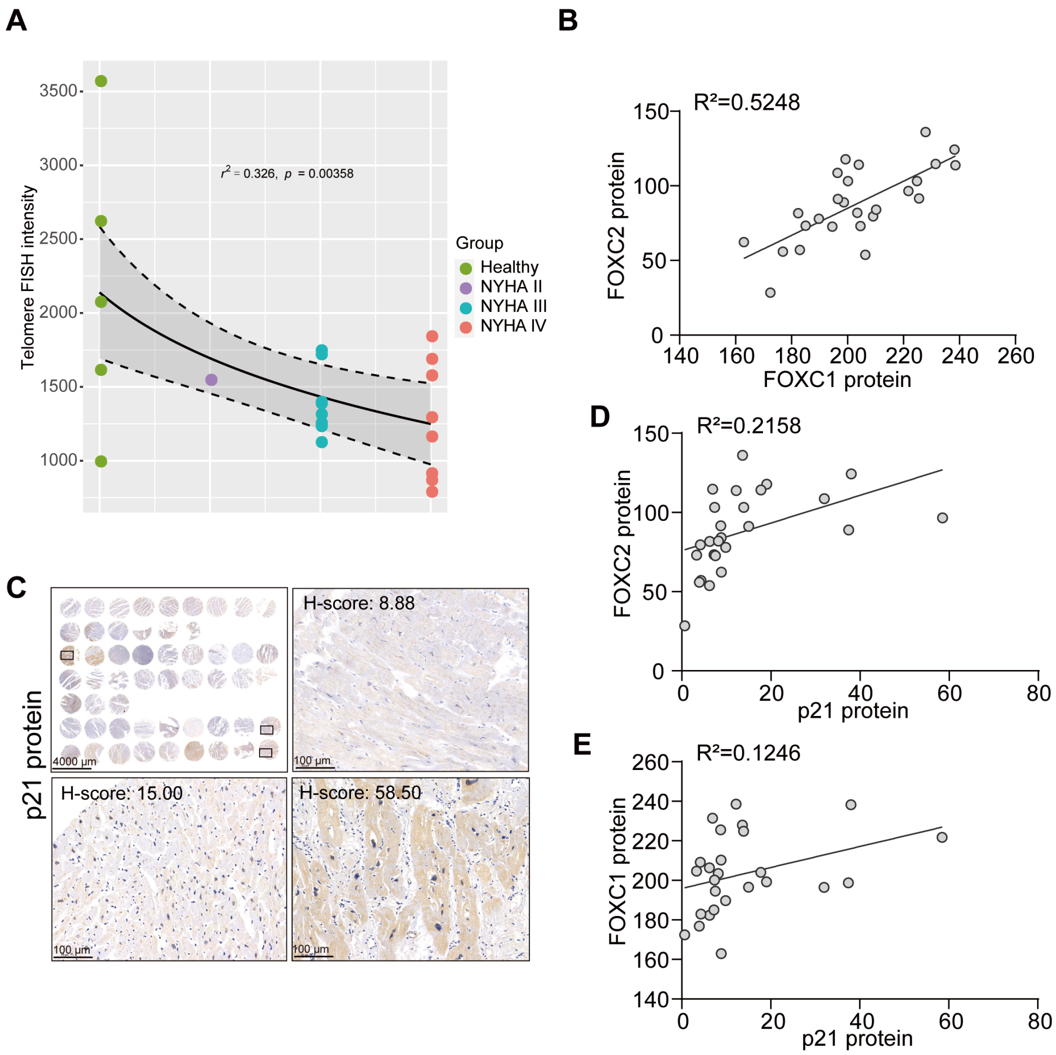

Figure S15: Correlation of FOXC1, FOXC2 and p21 protein levels in human heart biopsies. (A) Myocardial telomere lengths in healthy or heart failure (NYHA II-IV) biopsies. **(B)** FOXC1 and FOXC2 protein levels correlate positively in heart biopsies. **(C)** Representative images of p21 protein expression in heart failure biopsies. **(D)** FOXC2 and **(E)** FOXC1 protein levels correlate positively with p21 protein levels in heart biopsies.

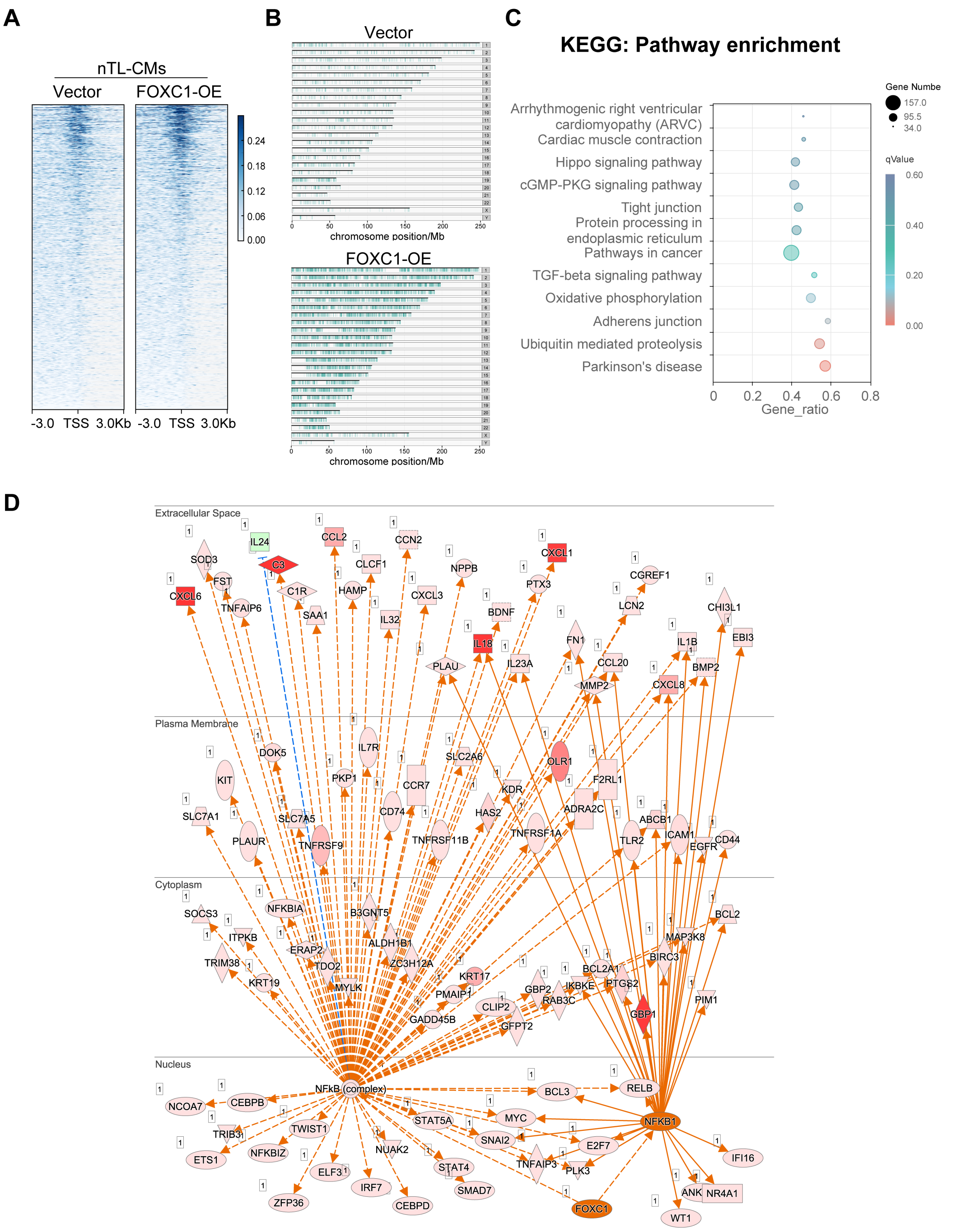

Figure S16: FOXC1-mediated transcriptional network in cardiomyocytes. (A) FOXC1 targets were captured using CUT & Tag assayed in vector or FOXC1 OE nTL-CMs. FOXC1 binding peaks within TSS ± 3Kb are shown. **(B)** Distribution of FOXC1 binding sites across chromosomes are shown. **(C)** KEGG analysis of enriched peaks in FOXC1 OE nTL-CMs. **(D)** IPA analysis of FOXC1 related genes.

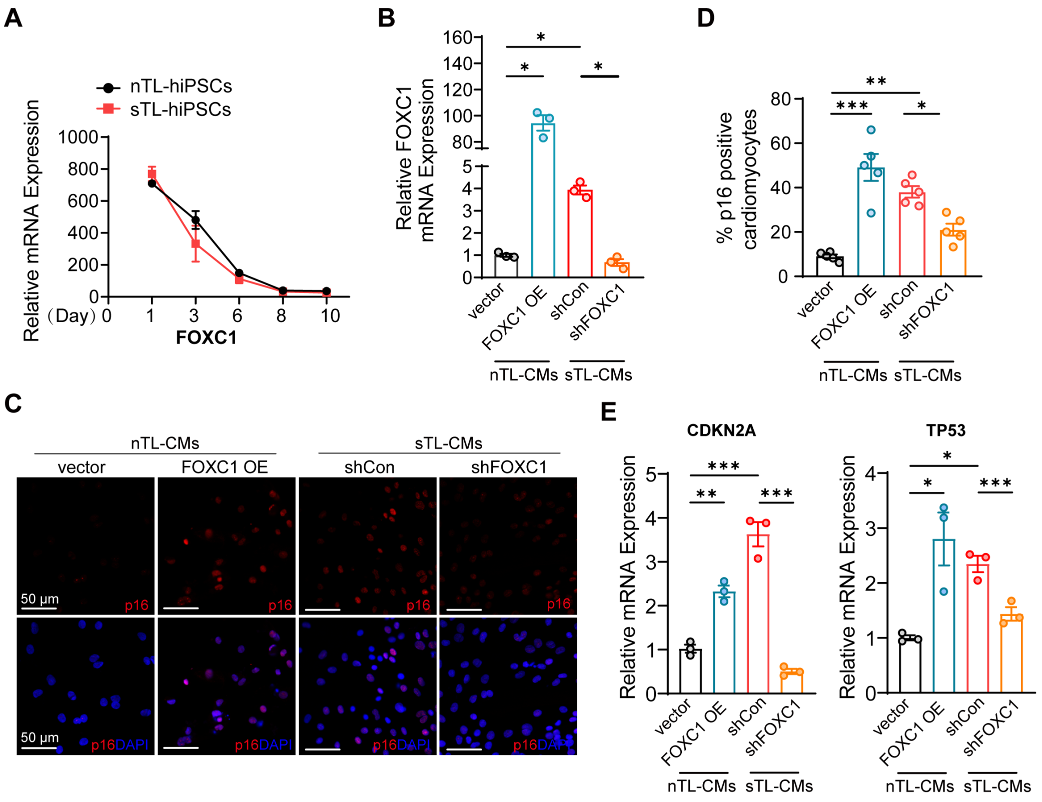

Figure S17: FOXC1 overexpression drives cardiac senescence and dysfunction in hiPSC-CMs. (A) Expression of FOXC1 during nTL- and sTL-hiPSCs cardiac differentiation (biological, n = 4 for each group). **(B)** FOXC1 overexpression (FOXC1 OE) in nTL-CMs and FOXC1 shRNA knockdown (shFOXC1) in sTL-CMs by lentivirus was evaluated by by RT-qPCR (biological, n = 3). **(C-D)** P16 positive cardiomyocytes in vector or FOXC1 OE treated nTL-CMs and shCon or shFOXC1 treated sTL-CMs (n = 107, 120, 143 and 152 cells, respectively, biological, n = 5 for each group). **(E)** Relative expression of senescence genes after FOXC1 overexpress or down-regulated (biological, n = 3 for each group). *$P \leq 0.05$, **$P \leq 0.01$ and ***$P \leq 0.001$. Data are presented as mean values ± SEM.

	Batch	Total Reads	Side 1 Aligned	Side 2 Aligned	Both Sides Aligned	Same Fragment	Dangling Ends
**nTL-CMs**	replicate 1	651544303	531733064	533239738	413428499	85389108	84229247
	replicate 2	681920159	554656060	554541924	427277825	63797798	63012182
**sTL-CMs**	replicate 1	568451905	459597033	462258128	353403256	74260228	73802614
	replicate 2	508857978	414556441	414270184	319968647	86857813	86226165
	**Batch**	**Self Circles**	**Error Pairs**	**cis Pairs**	**% cis Pairs**	**Unique Valid Pairs**	**% Unique Valid Pairs**
**nTL-CMs**	replicate 1	1117109	42752	91945533	37.73%	243673293	58.94%
	replicate 2	748231	37385	104347961	39.55%	263855455	61.75%
**sTL-CMs**	replicate 1	416925	40689	110189559	57.22%	192584092	54.49%
	replicate 2	581359	50289	87840954	55.72%	157655497	49.27%

Table S1. Mapping statistics and quality of Hi-C datasets.

Primer	Forward	Reverse
T	AAGGAGCTCACCAATGAGATG	TGACTTTGCTGAAGGAGACG
ISL1	CTGTGCTGAACGAGAAGCAGC	GTAAGCCACCGTCGTGTCTCTC
EOMES	GTGCCCACGTCTACCTGTG	CCTGCCCTGTTTCGTAATGAT
TBX5	CTGTGGCTAAAATTCCACGAAG T	GTGATCGTCGGCAGGTACAAT
NKX2.5	GTGGAGCTGGAGAAGACAGAG	CTGGAACCAGATCTTGACCTGC
GATA4	ACCACAAGATGAACGGCAT	CGTGGAGCTTCATGTAGAGG
TNNT2	GGAGGAGTCCAAACCAAAGCC	TCAAAGTCCACTCTCTCTCCATC
ACTN2	GACTCTGTGCCCTCATCCAC	CGCAAAAGCGTGGTAGAAGC
MYH6	TCAGCTGGAGGCCAAAAGTAA AGGA	TTCTTGAGCTCTGAGCACTCGTC T
FOXC1	TGTTCGAGTCACAGAGGATCG	ACAGTCGTAGACGAAAGCTCC
FOXC2	CCTCCTGGTATCTCAACCACA	GAGGGTCGAGTTCTCAATCCC
SCN7A	CAATGCGGCTTCCATCTTGTG	ACGCAATCAATCAGGACACTAAT
ENO1	TGGTGTCTATCGAAGATCCCTT	CCTTGGCGATCCTCTTTGG
ATL3	ACAGCCAGTCAACTGTGAAAG	CCAGACGACCGTATTCTGTGA
CDKN2A	ATGGAGCCTTCGGCTGACT	GTAACTATTCGGTGCGTTGGG
TP53	CAGCACATGACGGAGGTTGT	TCATCCAAATACTCCACACGC
GAPDH	GGAGCGAGATCCCTCCAAAAT	GGCTGTTGTCATACTTCTCATGG

Table S2. Real-Time quantitative PCR (RT-qPCR) Primers.

Primer	Forward	Reverse
2q-TERRA	AAAGCGGGAAACGAAAAGC	GCCTTGCCTTGGGAGAATCT
6q-TERRA	TTCTGACGCTGCACTTGAAC	TAGTGTGGAAAGCGGGAAAC
7p-TERRA	CAATCTCGGCTCACCACAATC	GGAGGCTGAGGCAGGAGAA
10q-TERRA	AAAGCGGGAAACGAAAAGC	GCCTTGCCTTGGGAGAATCT
12p-TERRA	AGTACCACCGAAATCTGT	GAGTTGCGTTCTCTTCAG
13q-TERRA	CCTGCGCACCGAGATTCT	GCACTTGAACCCTGCAATACAG
15q-TERRA	GCGTGGCTTTGGGACAACT	TGCAACCGGGAAAGATTTTATT
17q-TERRA	GTCCATGCATTCTCCATTGATAAG	AGCTACCTCTCTCAACACCAAGAAG
19p-TERRA	TTCAGAGTACCACCGAAA	GTTCTCCTCAGCACAGAC
GAPDH	AGCCACATCGCTCAGACAC	GCCCAATACGACCAAATCC
TERRA-specific RT-primer	CCCTAACCCTAACCCTAACCCTAACCCTAA	
GAPDH-specific RT-primer	GCCCAATACGACCAAATCC	

Table S3. Primers used for TERRA RT-qPCR.
